# Genome-wide variability in recombination activity is associated with meiotic chromatin organization

**DOI:** 10.1101/gr.275358.121

**Published:** 2021-09

**Authors:** Xiaofan Jin, Geoff Fudenberg, Katherine S. Pollard

**Affiliations:** 1 Gladstone Institutes, San Francisco, California 94158, USA;; 2 Department of Quantitative and Computational Biology, University of Southern California, Los Angeles, California 90089, USA;; 3 University of California San Francisco, San Francisco, California 94143, USA;; 4 Chan-Zuckerberg Biohub, San Francisco, California 94158, USA

## Abstract

Recombination enables reciprocal exchange of genomic information between parental chromosomes and successful segregation of homologous chromosomes during meiosis. Errors in this process lead to negative health outcomes, whereas variability in recombination rate affects genome evolution. In mammals, most crossovers occur in hotspots defined by PRDM9 motifs, although PRDM9 binding peaks are not all equally hot. We hypothesize that dynamic patterns of meiotic genome folding are linked to recombination activity. We apply an integrative bioinformatics approach to analyze how three-dimensional (3D) chromosomal organization during meiosis relates to rates of double-strand-break (DSB) and crossover (CO) formation at PRDM9 binding peaks. We show that active, spatially accessible genomic regions during meiotic prophase are associated with DSB-favored loci, which further adopt a transient locally active configuration in early prophase. Conversely, crossover formation is depleted among DSBs in spatially accessible regions during meiotic prophase, particularly within gene bodies. We also find evidence that active chromatin regions have smaller average loop sizes in mammalian meiosis. Collectively, these findings establish that differences in chromatin architecture along chromosomal axes are associated with variable recombination activity. We propose an updated framework describing how 3D organization of brush-loop chromosomes during meiosis may modulate recombination.

The formation of crossovers during meiotic recombination is a highly orchestrated process, enhancing genetic diversity by allowing reciprocal exchange of genomic information to occur between parental chromosomes. Crossover formation also promotes proper segregation of homologous chromosomes (Baker et al. 1976), and errors in this process lead to chromosomal abnormalities such as aneuploidy, which are associated with negative health outcomes (Petronis 1999; Potapova and Gorbsky 2017). In mammals, crossovers are highly enriched (100-fold) in discrete ∼1- to 2-kb stretches along the genome, termed recombination hotspots (Paigen and Petkov 2010). These hotspots are in large part determined by the binding of PRDM9, a meiosis-specific zinc-finger protein that marks loci for potential recombination (Baudat et al. 2010; Myers et al. 2010; Parvanov et al. 2010).

Although hotspot initiation is dependent on PRDM9, subsequent DSB and crossover formation are highly stochastic. Although exact numbers vary by species, a mammalian chromosome may harbor hundreds of PRDM9 binding loci, but during a typical meiotic cycle, only 10–20 double-stranded breaks (DSBs) occur (Diagouraga et al. 2018) per chromosome. Out of these DSBs, most are repaired as noncrossover conversion events, and only one or two per chromosome are chosen for crossover formation in mice (Baudat and de Massy 2007; Li et al. 2019). Local chromatin features such as GC content, histone modification, and cofactor binding are known to impact DSB formation at hotspots (Walker et al. 2015; Yamada et al. 2017), whereas nucleosome occupancy, GC content, and chromosomal position are associated with crossover formation (Hinch et al. 2019). Still, a full understanding of why certain hotspots are favored to form DSBs and crossovers remains undetermined.

Meiotic chromosomes adopt a brush-loop conformation characterized by chromatin loops attached to a central axis (Møens and Pearlman 1988). Although recombination hotspots are found within loops, DSB machinery, such as DNA-repair proteins, resides on the axis (Blat et al. 2002; Grey et al. 2018; Tock and Henderson 2018; Slotman et al. 2020). This “tethered-loop/axis complex” model of recombination suggests that 3D genome folding could place constraints on the recombination process. Here we apply computational analyses to investigate how 3D chromatin organization relates to PRDM9 binding, DSBs, and crossover formation in male mammalian meiosis. Our analyses aim to integrate observations from multiple recent interphase and meiosis data sets measuring recombination activity and chromatin organization, including Hi-C, leading to an updated framework of how meiotic events related to recombination are associated with brush-loop chromosomal architecture.

## Results

**Figure 1.**
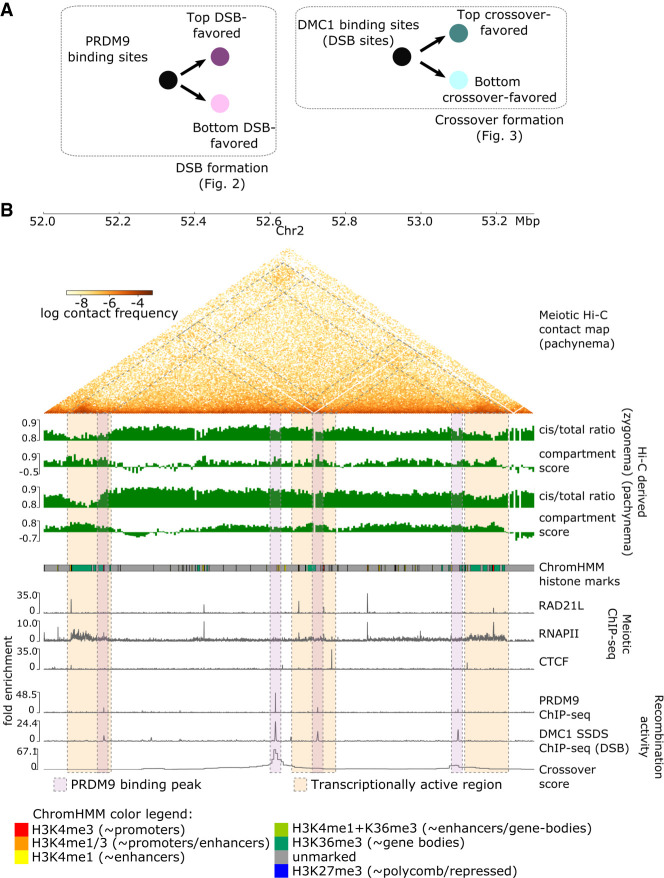
Multiple chromatin organization data sets are integrated with measurements of recombination activity at the levels of PRDM9 binding, DSB, and crossover formation. (*A*) Overview of recombination-related comparisons explored in this paper. Figure 2 explores chromatin conformation at PRDM9 binding peaks, stratifying based on their likelihood of forming DSBs as measured via DMC1-SSDS ChIP-seq signal. Subsequently, Figure 3 explores chromatin conformation at DMC1-SSDS binding peaks, indicative of DSBs, stratifying based on their likelihood of forming crossovers. (*B*) Key data sets used in this study (see also Supplemental Section S1), shown in a browser view of a representative 1.3-Mb region on mm10 Chromosome 2. Pachynema Hi-C contact frequencies are shown as a heatmap, in addition to Hi-C-derived cis/total ratio and compartment score for zygonema and pachynema. Hi-C contact information is accompanied by epigenetic chromatin state information using ChromHMM annotations of histone marks in mouse testis (for color legend, see *bottom*), as well as meiotic ChIP-seq tracks of cohesin subunit RAD21L, RNAPII, and CTCF. Recombination activity measurements include ChIP-seq binding tracks of PRDM9, DMC1 (marking DSBs), as well as crossover likelihood score derived from single-sperm whole-genome sequencing. Several relationships to note in this region: (1) enriched Hi-C contacts between transcriptionally active regions during meiosis, highlighted in orange shaded boxes; (2) colocalized DSB formation and crossover formation at PRDM9 binding peaks, highlighted in purple shaded boxes; (3) differences in DSB and crossover likelihood among PRDM9 binding peaks; and (4) locally depressed cis/total ratio and elevated compartment score at these loci in zygonema. Hi-C bins with missing data are ignored for visualization of maps and derived scores.

To investigate the relationship between meiotic chromatin structure and male recombination in the mouse genome, we first quantify recombination activity by analyzing PRDM9 binding measured by ChIP-seq (Baker et al. 2015), DSB activity based on DMC1 single-stranded DNA sequencing ChIP-seq (DMC1-SSDS) (Smagulova et al. 2016), and crossover likelihood quantified in single-sperm genome sequencing data sets (Yin et al. 2019). These three data sets all derive from a B6xCAST hybrid mouse genotype, allowing separate analyses of how PRDM9 binding yields DSBs and how DSBs are selected to form crossovers. Therefore, we explore chromatin features associated with (1) PRDM9 binding peaks, additionally partitioned between DSB-favored and disfavored, as well as (2) DMC1-SSDS binding peaks marking DSBs, additionally partitioned between CO-favored and disfavored (Fig. 1A).

We next integrate data sets quantifying meiotic chromatin structure. We analyze meiotic chromosomal structure using Hi-C contact maps of mouse spermatocytes in the zygonema and pachynema stages of prophase I. Although multiple meiotic Hi-C spermatocyte data sets have been published recently (Alavattam et al. 2019; Vara et al. 2019; Wang et al. 2019; Luo et al. 2020), we primarily focus on one that uses a B6xCAST genotype (Patel et al. 2019), matched with recombination data. In addition to raw contact frequencies, we also use Hi-C data to generate several genome-wide measures of meiotic chromatin 3D structure: cis/total ratio, A- and B-compartment scores, insulation scores, and FIRE scores. We highlight primarily the first two measures, which display particularly interesting patterns related to recombination. Cis/total ratios quantify the fraction of contacts within versus between chromosomes. Low cis/total ratios are associated with increased spatial accessibility (not to be confused with DNA accessibility associated with nucleosome occupancy) (Kalhor et al. 2012). At a chromosome-wide level, lower cis/total ratios indicate a greater degree of chromosome territoriality (Falk et al. 2019). A- and B-compartment scores quantify preferential interactions after removing the impact of genomic distance; positive compartment scores (A-compartment) are typically associated with active, gene-rich chromatin (Lieberman-Aiden et al. 2009). Meanwhile, insulation scores typically show minima at domain boundaries (Crane et al. 2015), whereas high FIRE scores indicate regions with enriched interactions (Schmitt et al. 2016). We supplemented these Hi-C metrics with measurements of chromatin state, including occupancy patterns of CTCF, cohesin (using the meiotic-specific cohesin subunit RAD21L—similar occupancy patterns exist for REC8) (see Supplemental Fig. S3A; Vara et al. 2019), and RNA polymerase II (RNAPII) (Margolin et al. 2014). We also include in our analysis a seven-state ChromHMM genomic profile from mouse testis (see Methods, “ChromHMM Chromatin Epigenetic States”) (Yue et al. 2014). All data sets are uniformly mapped to a consistent set of 5-kb bins across the autosomal chromosomes (492,557 bins genome-wide) (Fig. 1B). Meiotic data sets are compared with counterparts (Hi-C, CTCF, RNAPII, and cohesin RAD21 subunit) from embryonic stem (ES) cells, serving as an example of interphase chromatin organization (Nitzsche et al. 2011; Shen et al. 2012; Bonev et al. 2017), allowing the identification of meiosis-specific patterns.

### PRDM9 sites associated with DSB formation show transient shifts toward active, spatially accessible chromatin

**Figure 2.**
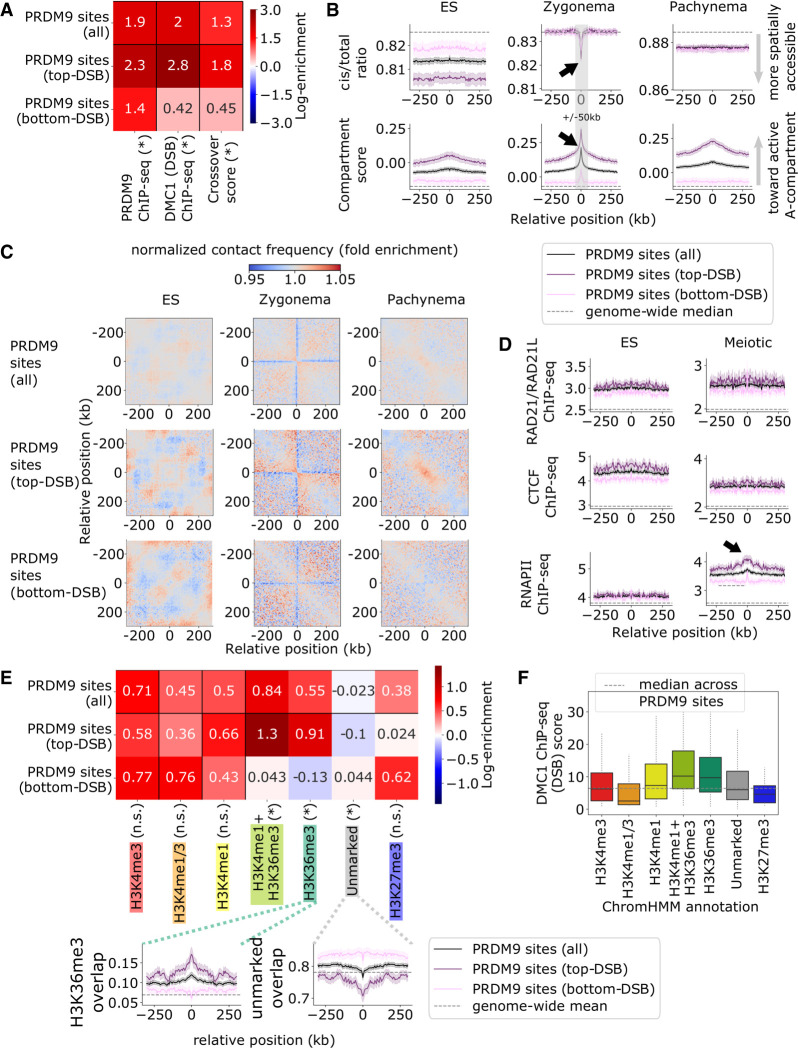
Chromatin environments at PRDM9 sites. (*A*) Summary of recombination activity at PRDM9 sites, with additional partition into the top and bottom quartiles by DMC1-SSDS ChIP-seq score, measuring DSB activity. Top (i.e., DSB-favored) sites have more bound PRDM9 and greater likelihood of crossover formation. Heatmap shows log fold enrichment over genome median, and an asterisk indicates a Bonferroni-adjusted *P* < 0.01 difference between top and bottom partitioned sites. (*B*) Hi-C cis/total ratio (*top*) and compartment score (*bottom*), symmetric-averaged across PRDM9 sites, calculated for ES, zygonema, and pachynema data sets. Shading represents 95% confidence intervals. Top DSB-favored sites are associated with higher compartment score in all data sets and lower cis/total ratio in ES. Black arrows indicate zygomena-specific shifts toward active, spatially accessible chromatin, which are enhanced at DSB-favored sites. (*C*) Normalized chromatin contact matrices, symmetric-averaged across PRDM9 sites, for embryonic stem (ES) cell, zygonema, and pachynema Hi-C data sets. We observe contact depletion at PRDM9 sites during zygonema and enriched contacts near DSB-favored sites during pachynema. (*D*) RAD21/RAD21L cohesin subunit (*top*), CTCF (*middle*), and RNAPII (*bottom*) ChIP-seq tracks, symmetric-averaged across PRDM9 sites, calculated for ES cell and meiotic data sets. Elevated RNAPII occupancy during meiosis appears to be associated with increased DSB formation (black arrow). (*E*) Overlap of ChromHMM histone annotations with PRDM9 sites. Note DSB-favored sites are depleted for unmarked chromatin while enriched for H3K36me3 chromatin typical of gene bodies. Heatmap shows log fold enrichment over genome-wide mean, and an asterisk indicates a Bonferroni-adjusted *P* < 0.01 difference between most (*top*) and least (*bottom*) DSB-favored sites. *Insets* plot overlap fraction symmetric-averaged around PRDM9 sites; shading represents 95% confidence intervals. (*F*) Distribution of DMC1 ChIP-seq scores (i.e., DSB activity) at PRDM9 sites split by ChromHMM state. DSB formation is elevated within H3K36me3-marked chromatin characteristic of gene bodies.

We begin by exploring chromatin organization at PRDM9 sites, defined as bins that intersect a PRDM9 ChIP-seq binding peak. We additionally investigate features that affect the likelihood of DSB formation at PRDM9 sites by partitioning these sites into quartiles with the highest and lowest respective DMC1-SSDS ChIP-seq scores (i.e., the most and least favored PRDM9 sites for DSB formation) (Fig. 2A).

We confirm that PRDM9 sites are enriched for DSBs and crossovers (5636 genomic bins) (Fig. 2A) and that PRDM9 ChIP-seq peaks are found in both A and B-compartment (57%A vs. 43%B) (Supplemental Fig. S1C). During zygonema, PRDM9 sites are characterized by locally decreased cis/total ratio and elevated A/B-compartment scores (Fig. 2B) within a ±50-kb window. These transient zygonema-specific signals reflect active and spatially accessible chromatin, respectively, and temporally coincide with H3K4me3 trimethylation activity at PRDM9 binding peaks (Buard et al. 2009; Baker et al. 2014), which peaks in zygonema and fades in pachynema (Chen et al. 2020). Averaged together, PRDM9 sites also show distinctive features in their local Hi-C contact map specifically during zygonema (Fig. 2C). However, these meiotic Hi-C patterns are weaker than signals in interphase such as at RAD21 sites in ES cells (Supplemental Fig. S3F), consistent with the general attenuation of TAD and compartment patterns in meiotic Hi-C data sets (Patel et al. 2019).

Top quartile DSB-favored sites are characterized by strong PRDM9 binding (Fig. 2A) and also show stronger local shifts toward active, spatially accessible chromatin during zygonema relative to disfavored sites (Fig. 2B,C). This supports the idea that local active chromatin shifts observed during zygonema are related to PRDM9 methyltransferase activity, which is positively associated with DSB formation (Baker et al. 2014). In addition to local zygonema-specific effects, DSB-favored sites appear to be biased at a more global scale toward active and spatially accessible chromosomal regions throughout interphase and meiosis (Fig. 2B). We find that these DSB-favored sites also show higher meiotic RNAPII occupancy, whereas CTCF and RAD21/RAD21L cohesin subunit occupancy do not appear strongly associated (Fig. 2D). We find enrichment of H3K36me3 histone marks (characteristic of gene bodies) and depletion of unmarked chromatin at DSB-favored sites (Fig. 2E). Among all ChromHMM annotations, DSBs appear most favored at sites associated with H3K36me3 (Fig. 2F). These results indicate DSB formation is enriched among PRDM9 sites in active/spatially accessible chromatin such as gene bodies. Indeed, compared with PRDM9 ChIP-seq peaks, DMC1-SSDS peaks are more skewed to A- rather than B-compartment (66%A vs. 34%B) (Supplemental Fig. S1C).

Finally, as noted earlier, these results are derived from a B6xCAST hybrid mouse genotype. To confirm whether these patterns are universal to PRDM9 binding in general or are somehow unique to a particular PRDM9 allele or genome background (e.g., B6 vs. CAST), we reran the analysis using a PRDM9 ChIP-seq data set that separately analyzed binding of B6 and CAST PRDM9 alleles (Grey et al. 2017; Supplemental Fig. S1) and also used haplotype-resolved Hi-C (Patel et al. 2019; Supplemental Fig. S2) to distinguish between B6-B6, CAST-CAST, and interhomolog genomic Hi-C contacts. We confirmed that our overall conclusions hold for both the B6 and CAST PRDM9 allele and for B6-B6, CAST-CAST, and interhomolog Hi-C contacts, suggesting that our observations are general properties of meiotic recombination.

### Depleted crossover formation for DSB sites in spatially accessible chromatin, especially gene bodies

**Figure 3.**
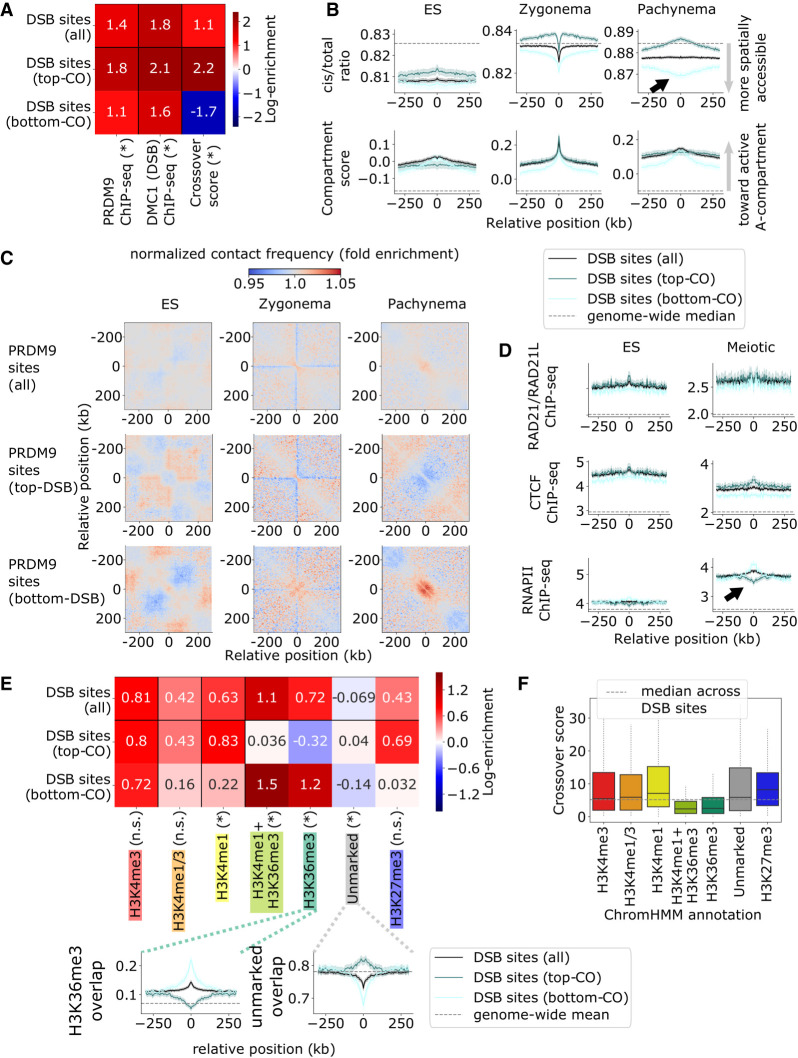
Spatially accessible chromatin is depleted for crossover formation, particularly at gene bodies. (*A*) Summary of recombination activity at DSB binding sites (from DMC1-SSDS ChIP-seq), with additional partition into the top and bottom quartiles for crossover likelihood. Top (i.e., crossover-favored) sites have modestly stronger inherent DSB activity and show greater likelihood of crossover formation. Heatmap shows log fold enrichment over genome median, and an asterisk indicates a Bonferroni-adjusted *P* < 0.01 difference between the top and bottom partitioned sites. (*B*) Hi-C cis/total ratio (*top*) and compartment score (*bottom*), symmetric-averaged across DSB sites, calculated for ES cell, zygonema, and pachynema data sets. Shading represents 95% confidence intervals. Top CO-favored sites are associated with a higher cis/total ratio, particularly during meiosis, indicative of reduced spatial accessibility (black arrow). (*C*) Normalized chromatin contact matrices, symmetric-averaged across DSB sites, for ES cell, zygonema, and pachynema Hi-C data sets. We observe reduced contact frequency near top CO-favored sites and vice versa, particularly during pachynema. (*D*) RAD21/RAD21L cohesin subunit (*top*), CTCF (*middle*), and RNAPII (*bottom*) ChIP-seq tracks, symmetric-averaged across DSB sites, calculated for ES cell and meiotic data sets. Elevated RNAPII occupancy during meiosis appears to be associated with decreased crossover formation (black arrow). (*E*) Overlap of ChromHMM histone annotations with crossover-partitioned DSB sites. Note CO-favored sites are depleted for the H3K36me3 chromatin typical of gene bodies and are enriched for unmarked chromatin. Heatmap shows log fold enrichment over genome-wide mean, and an asterisk indicates a Bonferroni-adjusted *P* < 0.01 difference between most (*top*) and least (*bottom*) CO-favored sites. *Insets* plot overlap fraction symmetric-averaged around DSB sites; shading represents 95% confidence intervals. (*F*) Distribution of crossover likelihood scores at DSB sites split by ChromHMM state. Crossover formation is depleted at DSBs in H3K36me3 chromatin, despite abundant DSB activity (see Fig. 2F), indicating that gene-body crossover depletion occurs at the DSB-to-CO stage rather than the PRDM9-to-DSB stage.

We next explore how chromatin organization around DSBs affects their likelihood of being selected as the site of crossover formation later during pachynema. We partition DSB sites (i.e., the 9569 bins intersecting DMC1-SSDS ChIP-seq peaks) into quartiles based on their crossover likelihood (Fig. 3A). We find that the top quartile of crossover-favored DSB sites are characterized by an elevated cis/total ratio throughout meiosis and interphase, as well as modestly lower compartment scores (Fig. 3B). These sites also show depleted nearby (±100 kb) Hi-C contacts (Fig. 3C) particularly during pachynema. This indicates that DSBs in chromosomal regions that adopt, on average, fewer spatially accessible configurations are favored for crossover formation, contrasting the clear enrichment of DSBs at PRDM9 sites in active, spatially accessible chromatin.

Higher meiotic RNAPII occupancy and elevated H3K36me3 ChromHMM overlap (characteristic of gene bodies) appear associated with decreased crossover activity (Fig. 3D,E). Depletion of crossover formation in H3K36me3 gene body regions is confirmed by comparing crossover scores at DSB sites across different ChromHMM histone annotations, validating that this gene-body recombination depletion is not occurring at the earlier stage of DSB formation (cf. Fig. 3F and Fig. 2F).

DSB sites that colocalize with H3K36me3 histone marks are characterized by a low cis/total ratio (i.e., high spatial accessibility), which is associated with reduced crossover formation. However, crossover depletion at low cis/total ratio sites is observed beyond H3K36me3 regions (Supplemental Fig. S4). This suggests that crossover formation is generally depleted in active and spatially accessible chromatin (gene bodies as a particular example) and that distinctly different chromatin environments favor DSB formation and crossover formation.

### Linear model with principal component analysis reveals recombination-associated chromatin features

To jointly assess the contributions of different chromatin structure variables to the likelihood of DSB formation at PRDM9 sites and crossover formation at DSB sites, we implement a linear modeling approach that uses principal component analysis (PCA) and model selection. These techniques help us to address the problem that Hi-C scores (cis/total ratio, compartment score, insulation, FIRE), epigenetic states (ChromHMM), and ChIP-seq signals (RAD21/RAD21L cohesin, CTCF, RNAPII) constitute a large collection of chromatin features, many of which are correlated with each other (Supplemental Fig. S5A).

**Figure 4.**
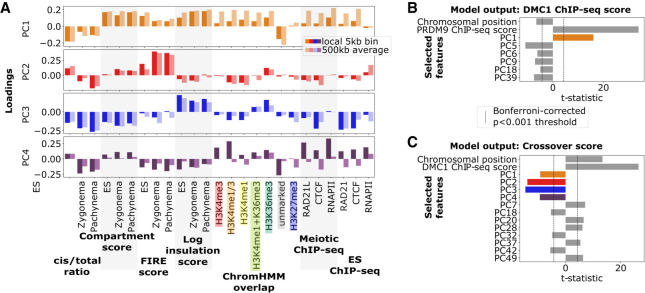
Principal component analysis (PCA) with linear model reveals variable chromatin organization at PRDM9 and DSB sites and its relationship with recombination activity. (*A*) Loadings for the top four principal components (PC1–PC4) of variation at joint PRDM9–DSB sites based on underlying chromatin organization variables (horizontal axis). Two values of each variable were included: one measuring local value at the joint PRDM9–DSB site and the other a 500-kb average around the site. Positive PC1 loadings reflect presence of active chromatin, which is typically characterized by a low cis/total ratio, high compartment, FIRE, and insulation scores, as well as increased histone modifications, RAD21L cohesin subunit, and RNAPII. Positive PC2 loadings indicate the presence of H3K36me3 histone marks typical of gene bodies, which tend to colocalize with increases in FIRE score, as well as meiotic-specific decreases in cis/total ratio. PC3 reflects divergence from the typical correlation between activity and spatial accessibility: Specifically, positive PC3 loadings indicate chromatin regions that are more spatially accessible (lower cis/total ratio) than expected given their activity (compartment score). Positive PC4 loadings indicate strong local enrichment of RAD21L/CTCF/RNAPII, characteristic of occupancy sites. (*B*) Results from a linear model for DSB activity (quantified by DMC1-SSDS ChIP-seq score) at PRDM9 sites as a function of the principal components described in *A* and adjusted for centromeric proximity (chromosomal position) and inherent PRDM9 binding variability (PRDM9 ChIP-seq score). Forward selection was used to choose statistically significant principal components to include in the model. Note that the strongest predictors are positive inherent PRDM9 binding strength and positive PC1, reflecting increased DSB formation at PRDM9 sites in active, spatially accessible chromatin. PC colors as in panel *A*. (*C*) Results from a linear model with forward feature selection for crossover likelihood at DSB sites as a function of principal components and adjusted for centromeric proximity (chromosomal position) and inherent DSB variability (DMC1-SSDS ChIP-seq score). DMC1-SSDS score and chromosomal position both positively predict crossover formation, reflective of inherent DSB variability and pericentromeric crossover depletion. PC1–PC4 all negatively predict crossover formation, reflecting crossover depletion at DSB sites in active chromatin, particularly at gene bodies, as well as promoters and spatially accessible regions. PC colors as in panel *A*.

First, we apply PCA to extract the primary directions of variation in chromatin variables from both ES and meiotic time points. To emphasize variation across PRDM9 and DSB sites, as opposed to genome-wide variation, we applied PCA among joint PRDM9–DSB sites, defined as the union of bins intersecting either PRDM9 or DMC1-SSDS ChIP-seq sites (Supplemental Fig. S5B). We find that the first principal component (PC1), encompassing by far the highest explained variance (16.0%), is positively associated with many of the general trends of active and spatially accessible chromatin. Increased levels of PC1 correspond with a lower cis/total ratio, higher compartment score, and increased active epigenetic marks (Fig. 4A). The second principal component (PC2; 3.7% explained variance) positively associates with the presence of gene bodies (H3K36me3), along with high Hi-C FIRE scores, and meiosis-specific decreases in the cis/total ratio. The third principal component (PC3; 2.9% explained variance) reflects instances in which the cis/total ratio and compartment scores diverge from their PC1 association. Positive PC3 indicates when spatial accessibility is higher than expected given chromatin activity. Finally, the fourth principal component (PC4; 2.5% explained variance) appears to reflect local enrichment of the meiotic RAD21L cohesin subunit, H3K4me3, and RNAPII, consistent with presence of active promoters.

After transforming chromatin variables into principal component space, we use a linear model with feature selection to identify statistically significant associations between principal components and either DSB or crossover formation. Applying this strategy to DSB formation at PRDM9 sites (Fig. 4B), we treat individual PRDM9 sites as quasi-independent observations. We include PRDM9 binding score and chromosomal position in the model to adjust for inherent differences in PRDM9 binding and potential centromeric or telomeric proximity effects (mouse chromosomes are acrocentric, meaning chromosomal position can be used as a proxy for centromeric distance). We find that PRDM9 binding strength and PC1 are both strong predictors of DSB formation. The positive coefficient for PC1 confirms that DSBs are enriched in active chromatin, even after adjusting for binding strength differences at PRDM9 sites. This result aligns with previous reports detailing increased DSB formation at PRDM9 sites in chromatin with prior histone modifications, as well as a balance between intrinsic affinity for PRDM9 binding and chromatin environment in determining DSB formation (Walker et al. 2015). Comparing explained variance between the naive model, only using PRDM9 ChIP-seq strength, and our selected model incorporating structural information, we find that R^2^ increases from 19.2% to 27.9%.

Next, we apply the same approach to modeling the distribution of crossover likelihood at DSB sites (Fig. 4C), including DMC1-SSDS ChIP-seq score as a predictor to adjust for inherent differences in DSB formation. We find that PC1 (chromatin activity) shows a negative association with crossover likelihood, confirming that active and spatially accessible chromatin is disfavored for crossovers, in contrast to its positive association with DSBs. Additionally, PC2–PC4 are also negative predictors of crossover likelihood, respectively, indicating that gene bodies, promoters, and extraspatially accessible chromatin are disfavored for crossover formation. We also find a strong positive correlation with chromosomal position as expected from crossover suppression near centromeres (Blitzblau et al. 2007; Nambiar and Smith 2016). Chromosomal position (along chromosomal arm) was only selected in the crossover prediction model (Fig. 4C) and not in the DSB model (Fig. 4B), supporting earlier conclusions that pericentromeric regions are depleted for crossovers but not DSBs (Blitzblau et al. 2007; Talbert and Henikoff 2010; Vincenten et al. 2015). Comparing explained variance between a naive model—only using DMC1 ChIP-seq strength and chromosomal position—and our selected model incorporating structural information, we find that *R*^2^ increases from 10.9% to 19.8%.

Finally, although our analysis here separately assesses two stages of recombination (PRDM9-to-DSB and DSB-to-crossover), we also apply our approach to analyze PRDM9 sites tracked all the way through DSB formation and into crossover formation (i.e., PRDM9-to-crossover). This analysis (Supplemental Fig. S6) indicates that high spatial accessibility (low meiotic Hi-C cis/total ratio) and overlap with gene bodies are negative determinants of crossover formation at PRDM9 sites but that overall crossovers are still favored in the A-compartment rather than B-compartment. This observation that crossovers remain enriched in active A-compartment chromatin despite depletion in gene bodies also helps place our findings in the context of earlier work reporting positive genome-wide correlations between gene bodies and crossovers (Yin et al. 2019). A positive correlation would be expected owing to gene bodies also being enriched in A-compartment, an expectation we confirm when calculating global genome-wide correlation between H3K36me3 ChromHMM overlap and crossover score; however, a localized correlation analysis considering only PRDM9 and DSB sites reveals a negative correlation between H3K36me3 and crossover formation (Supplemental Fig. S7), consistent with our linear modeling approach.

### Organization of A- and B-compartment genomic regions show different chromosomal loop lengths

**Figure 5.**
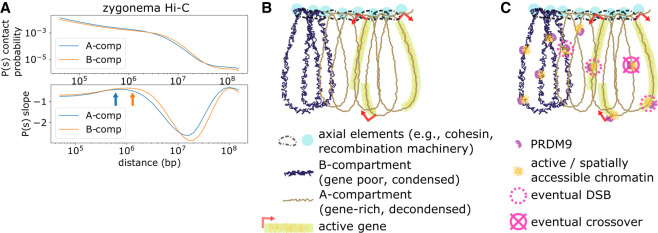
Proposed framework relating mammalian meiotic chromosomal architecture and recombination. (*A*) Contact probability versus genomic distance analysis of zygonema Hi-C data set. Orange and blue arrows indicate estimate of loop length for A- and B-compartments, respectively, determined as the maxima of the derivatives of the *P*(*s*) curves as in Gassler et al. (2017). (*B*) Simplified cartoon of proposed chromatin conformation. In early leptonema, meiotic chromosomes adopt a brush-loop architecture, with cohesin and recombination machinery located at the axis. Loops in the A-compartment have, on average, fewer base pairs than the B-compartment. Accordingly, A- and B-compartment regions depicted here represent roughly equal genomic lengths despite greater number of A-compartment loops. Physical size of A- and B-compartment loops may remain comparable owing to the relaxed linear packing density in A-compartment. (*C*) Concurrently during leptonema, PRDM9 binds across both A- and B-compartment regions, causing local increases in chromatin activity and spatial accessibility. Schematic depicts hypothetical example of 10 total binding events across the A- and B-compartment regions. After PRDM9 binding, a subset of binding loci are recruited to DSB machinery at the axis and form DSBs. This subset is biased toward A-compartment. Later during pachynema, a single crossover point is selected from the DSBs formed earlier, avoiding DSBs formed in gene-body regions.

Given the importance of chromatin activity and spatial accessibility in recombination, we further explored how A- and B-compartment regions are organized in the context of brush-loop meiotic chromosomes. By analyzing contact frequency versus distance curves (Gassler et al. 2017) in meiotic Hi-C data, we find the maxima of the derivatives occur on average approximately threefold shorter in the A-compartment compared with the B-compartment during zygonema (Fig. 5A). Similar results are observed in pachynema and other meiotic Hi-C data sets, including the DSB-deficient *Gm960^−/−^* (also known as *Top6bl^−/−^*) genotype, but not in mitotic Hi-C (Supplemental Fig. S8). This suggests that meiotic loops in the A-compartment contain approximately threefold fewer base pairs than the B-compartment, implying a higher frequency of loop-axis attachment points in active A-compartment chromatin. This would align with previous cytological analysis suggesting that active chromatin regions are overrepresented along the axis (i.e., stretched relative to inactive chromatin) (Luciani et al. 1988; Fransz et al. 2000).

We observe an enrichment of cohesin binding sites from published ChIP-seq data sets (Nitzsche et al. 2011; Vara et al. 2019) in the A-compartment compared with the B-compartment, particularly in meiosis-specific subunits RAD21L/REC8 (Supplemental Fig. S3A). This finding appears to align with our hypothesis, as cohesin is known to localize at the axes of meiotic chromosomes. However, we note that meiotic cohesin binding sites are characterized by a low cis/total ratio, indicating a high degree of spatial accessibility not expected at chromosomal axes, and also show a high degree of overlap with promoter regions (Vara et al. 2019; Supplemental Fig. S3C). Therefore, we refrain from interpreting meiotic cohesin REC8/RAD21L binding sites as marking stable sites of axial localization along the chromosome; the possibility of off-axis cohesin binding has also been previously noted (Vara et al. 2019).

As a caveat to our prediction of threefold shorter A-compartment loops, we note that the physical size of the meiotic loops appears relatively consistent overall along chromosomal axes. Although physical loop size differences can be observed near telomeres and with exogeneous DNA (Heng et al. 1996; Zickler and Kleckner 1999; Kolas et al. 2004), differences between active and inactive chromatin (e.g., A- vs. B-compartment) have not been reported to our knowledge. We hypothesize that relative decondensation of A-compartment chromatin fibers may compensate for the fewer base pairs in A-compartment loops, leading to comparable physical sizes between A/B-compartment loops (Fig. 5B). Enrichment of axial loci in active, decondensed chromatin may partially explain the otherwise puzzling low cis/total ratio at meiotic cohesin REC8/RAD21L ChIP-seq sites, given that active, decondensed chromatin is generally associated with a lower cis/total ratio (Mahy et al. 2002; Kalhor et al. 2012).

## Discussion

We test the hypothesis that features of 3D genome organization are associated with meiotic recombination in the mammalian genome. Our analyses reveal distinct associations with PRDM9 binding, DSB formation, and crossover formation. We confirm these observations with multiple regression and analyze contact frequency decay to help situate these events relative to the meiotic brush-loop structure (Fig. 5B,C). This work complements earlier multifactorial analysis of DSB and crossover formation at hotspots (Walker et al. 2015; Yamada et al. 2017; Hinch et al. 2019) by showing that in addition to local chromatin environment, features of genome folding beyond the immediate vicinity of hotspots (e.g., >5 kb) are significantly associated with differences in recombination. Based on the results of our linear model, we estimate that incorporation of these chromatin data sets improves prediction of DSB formation at PRDM9 sites by nearly a factor of 1.5 (model *R*^2^ improves from 19.2% to 27.9%) and our ability to predict crossovers at DSB sites nearly twofold (model *R*^2^ improves from 10.9% to 19.8%).

PRDM9 sites are locally associated with a transient shift toward increased compartment scores and reduced cis/total ratios during zygonema. This builds on previous observations of a simultaneous transient shift in patterns of histone trimethylation (e.g., H3K4me3) around PRDM9 binding in mammals (Baudat et al. 2010; Myers et al. 2010; Parvanov et al. 2010; Chen et al. 2020).

We find DSB formation at PRDM9 sites is strongly associated with elevated compartment scores during meiosis and interphase, characteristic of active chromatin, and lower cis/total ratio during interphase, characteristic of spatially accessible chromatin. We confirm DSB enrichment in active (A-compartment) chromatin (Patel et al. 2019). DSB formation is also favored in PRDM9 sites with enhanced zygonema-specific shifts toward increased activity and spatial accessibility.

In contrast to DSB formation at PRDM9 sites, crossovers are favored at DSB sites with increased cis/total ratios on average, indicating less spatially accessible chromatin. DSB sites in spatially accessible gene body regions marked by H3K36me3 are particularly depleted for crossovers, consistent with earlier reports in plants (Wijnker et al. 2013). Crucially, our analysis discerned this relationship by focusing on DSB sites. By comparison, because of limited resolution of crossover locations, positive correlations can be observed genome-wide between genes and crossover frequency (Yin et al. 2019) as both crossovers and gene bodies are enriched in active chromatin. Our results indicate that crossover depletion exists in gene bodies and occurs at the crossover selection stage rather than during DSB formation. This may be a general feature of 3D genome structure, as decreased Hi-C cis/total ratio is associated with crossover depletion among DSB sites beyond H3K36me3 regions as well.

Our observations that DSB formation is enriched in active, A-compartment chromatin, including gene bodies, align with earlier work that showed DSB bias toward genic regions with active histone modifications and away from inactive chromatin regions such as lamin-associated regions (Smagulova et al. 2011; Walker et al. 2015; Patel et al. 2019). On the other hand, crossover depletion in spatially accessible genomic regions, such as gene bodies, is a more unexpected result: Why do meiotic cells create so many DSBs in these regions, only to almost never select them as eventual crossover locations? We speculate a potential causal mechanism for crossover depletion may involve reduced frequency of interhomolog contacts in transcriptionally active regions. RNA polymerase activity may disrupt interhomolog engagement and thus decrease the stability of recombination intermediates. Further experimental work is needed to test this hypothesis, although preliminary analysis using haplotype-resolved Hi-C indicates a decrease in interhomolog versus intra-homolog contacts in active H3K36me3 chromatin regions, and increased interhomolog versus intra-homolog contacts at crossover-favored DSB sites (Supplemental Fig. S9).

Because crossovers reflect rare and contingent events, interpreting ensemble-average Hi-C data sets presents many caveats. In any given cell, only a fraction of PRDM9 binding loci is converted to DSBs, and yet a smaller subfraction is selected as crossovers. Therefore, the Hi-C signals we observe at DSB or crossover-favored genomic sites are unlikely to directly reflect chromosomal configuration of individual DSB and crossover events, as even the most favored sites are not sites of recombination in most cells. Additionally, we note that genomic positions of DSBs and meiotic cohesin ChIP-seq sites appear to have relatively low cis/total ratios, despite the fact that immunofluorescent microscopy shows DSB machinery and cohesin subunits enriched along the axes (Ishiguro et al. 2011; Lee and Hirano 2011; Hinch et al. 2020). In general, genomic loci with axial positions in a brush-loop structure would be expected to display high cis/total ratios characteristic of low spatial accessibility. This puzzling observation is in contrast with yeast meiosis, where REC8 ChIP-seq sites display elevated cis/total ratios (Muller et al. 2018; Schalbetter et al. 2019), as expected for axially positioned loci in a loop-brush structure.

Our analyses of contact frequency curves in meiosis suggest that, on average, loops in active chromatin have fewer base pairs than in inactive chromatin. We speculate that overall linear packing density of chromatin may be higher in the B-compartment, compensating for base-pair length differences. This proposal is congruent with previously reported overrepresentation of A-compartment regions along chromosomal axes (Luciani et al. 1988; Fransz et al. 2000) and aligns with the enrichment of meiotic cohesin peaks in the A-compartment (Vara et al. 2019; Luo et al. 2020). Transcriptionally active genomic regions with decondensed chromatin are furthermore associated with increased spatial accessibility (Mahy et al. 2002; Kalhor et al. 2012); together this may resolve the otherwise puzzling low cis/total ratio observed at meiotic cohesin ChIP-seq sites, which often overlap promoters. Nevertheless, this association between transcriptional activity and axial localization requires a cautious interpretation. Despite sharing many ChIP-seq sites with cohesin at promoters, RNAPII, which is additionally enriched in gene bodies, is broadly dispersed along chromosomal loops (van der Laan et al. 2004). H3K4me3, which marks both promoters and PRDM9 binding loci during meiosis, is present both at axial and loop positions (Prakash et al. 2015), whereas H3K27me3, which typically associated with transcriptionally repressed promoters, localizes close to the axis (Prakash et al. 2015). Given these findings, we cannot currently rule out the possibility that cohesin is more uniformly distributed across active and inactive chromatin yet is preferentially visible in transcriptionally active regions such as promoters owing to hyper-ChIPability (Teytelman et al. 2013; Jain et al. 2015).

We conclude by suggesting avenues for future experimental studies. First, modern microscopy methods such as DNA-paint and super-resolution FISH (Beliveau et al. 2015; Schnitzbauer et al. 2017; Albert et al. 2019) would be useful to trace contiguous DNA regions and obtain direct evidence for differing numbers of base pairs per loop in active and inactive regions. If these experiments could be performed in conjunction with crossover tracking, it would also be interesting to explore hypotheses that differences in loop sizes may affect crossover interference (Paigen and Petkov 2010). Second, our results thus far largely show associations between genome organization and recombination; perturbation experiments would allow for analyses of causality. For instance, to test the effects of chromatin activity and spatial accessibility, we envision experiments that target the expression of genes near or overlapping recombination hotspots using CRISPR inhibition/activation tools (Larson et al. 2013; Maeder et al. 2013), in conjunction with chromosomal conformation capture and ChIP-seq experiments to observe downstream effects on meiotic genome folding, PRDM9 binding, DSB formation, and crossovers. Third, to explore the connection between PRDM9 methyltransferase activity and transient shifts in 3D genome organization, we imagine potential Hi-C and ChIP-seq experiments using heterozygous PRDM9 mutants with modified methyltransferase activity (Thibault-Sennett et al. 2018). Alternatively, CRISPR-based epigenome modification strategies (Hilton et al. 2015) can be repurposed to directly perturb histone marks around hotspots, exploring, for example, whether targeted local H3K4me3 deposition is sufficient to drive genome folding changes during meiosis to the extent observed in zygonema with PRDM9. Similar approaches could be used to investigate the effects of modified H3K36me3 levels for DSB and crossover formation. Fourth, our results are limited to male meiosis, and it remains to be seen whether similar conclusions can be made for female meiosis; confirmation would require analogous data sets generated during oogenesis. Finally, meiotic Hi-C for human chromosomes would enable an analysis of the potential interplay between *PRDM9* polymorphism (Baudat et al. 2010) and clinically relevant dysregulation of 3D genome folding in meiosis.

## Methods

All data sets pertaining to this manuscript are previously published and described in more detail in Supplemental Section S1.

### Genome mapping and 5-kb bins

All analyses were performed with the mouse mm10 genome assembly. Cooler makebins (Abdennur and Mirny 2020) was used to generate a 5-kb bin BED file corresponding to the mm10 genome. Using this BED file as auxiliary input, BEDTools intersect (Quinlan and Hall 2010) was used to convert unbinned genomic tracks to 5-kb resolution, outputting the maximum score and coverage per bin for bedGraph and BED tracks, respectively.

### ChIP-seq data sets

The ENCODE ChIP-seq pipeline (github.com/ENCODE-DCC/chip-seq-pipeline2) was used to process raw ChIP-seq reads into bigWig signal (fold change over input) and peak files (idr optimal) for ChIP-seq of PRDM9 (Baker et al. 2015; Grey et al. 2017), DMC1 (Smagulova et al. 2016), RNAPII (Shen et al. 2012; Margolin et al. 2014), CTCF (Nitzsche et al. 2011; Vara et al. 2019), RAD21 (Nitzsche et al. 2011), RAD21L, and REC8 (Vara et al. 2019). UCSC bigWigToBedGraph (Kent et al. 2010) was used to convert bigWig to bedGraph format before mapping to 5-kb bins. Binding sites were defined as genomic bins intersecting ChIP-seq peak centers.

### Partitioning PRDM9 and DSB sites into top and bottom quartiles by DSB/crossover activity, respectively

Considering all genomic bins corresponding to PRDM9 peaks, bins were ranked based on their DMC1-SSDS ChIP-seq score. The top and bottom ranked bins become the top-DSB and bottom-DSB PRDM9 bins, respectively. The same approach is used with genomic bins corresponding to DSBs, using the crossover score to rank.

### Generating genetic map of crossover scores from single-sperm-seq data set

A 5-kb resolution crossover score map was generated using a list of mapped crossovers from Yin et al. (2019). These loci were derived using single sperm sequencing of B6xCAST hybrid mouse (Yin et al. 2019). The crossover score for each 5-kb bin was determined by summing the number of intersecting crossovers, inversely weighted by the length of each crossover (i.e., giving prominence to sharply localized crossovers). The final crossover score was normalized by genome-wide median. Supplemental Figure S10 shows a visual summary of approach.

### ChromHMM chromatin epigenetic states

Chromatin state was obtained from the Mouse ENCODE Project, using the segmentation outputs from the testis-specific seven-state ChromHMM model (Yue et al. 2014). This mouse ENCODE data set used four histone modification measurements (H3K4me1, H3K4me3, H3K36me3, H3K27me3) to generate a seven-state model. The seven states are H3K4me3 ∼ promoters, H3K4me1/3 ∼ promoters/enhancers, H3K4me1 ∼ enhancers, H3K4me1 + H3K36me3 ∼ enhancers/gene-bodies, H3K36me3 ∼ gene-bodies, unmarked, and H3K27me3 ∼ repressed/polycomb. Coordinates were lifted over to mm10 from mm9 using UCSC liftOver (Hinrichs et al. 2006).

### Hi-C analysis

Raw Hi-C reads from ES cells (Bonev et al. 2017) and meiosis (zygonema, pachynema) (Patel et al. 2019; Vara et al. 2019) were converted using the HiCUP pipeline (Wingett et al. 2015) and cooler (Abdennur and Mirny 2020) software into cooler format at 5-kb resolution. Insulation scores and cis/total contacts were derived using cooltools.insulation and cooltools.coverage (github.com/open2c/cooltools, https://doi.org/10.5281/zenodo.4667696), respectively.

FIREcaller (Crowley et al. 2021) was used to generate FIRE scores. cooltools.expected was used to generate contact frequency versus genomic distance plots (i.e., *P*(*s*) curves), masking all A-compartment bins for the B-compartment analysis and vice versa, with compartments defined using the ES Hi-C data set.

### Compartment vector calling using a “fine-grain eigenvectors” approach

Although compartment vectors for ES Hi-C samples were successfully generated using cooltools.eigdecomp for whole chromosomes (i.e., traditional eigenvector decomposition), the approach failed to generate reasonable compartment vectors for several chromosomes in the pachynema and zygonema data set owing to the dropoff in signal of the Hi-C matrices beyond 10-Mb contact distances. Therefore, we implemented the “fine-grain eigenvector” approach presented by Wang et al. (2019) that calculates eigenvectors using 10-Mb × 10-Mb chunks along the Hi-C contact map.

### Haplotype-resolved Hi-C

Haplotype-resolved Hi-C reads for pachynema and zygonema Hi-C data sets were kindly provided by the original investigators (Patel et al. 2019) and processed into contact matrices using cooler.

### Visualization and plotting

Seaborn (Waskom 2021) and Matplotlib (Hunter 2007) were used to generate meta-averaged Hi-C matrices and genomic tracks; 95% confidence intervals are calculated using Seaborn defaults with 1000-sample bootstrapping. Plots are symmetrized for upstream versus downstream directions. PyGenomeTracks (Lopez-Delisle et al. 2021) was used to generate example genome tracks at particular loci. Bins with minimal read counts are filtered out by cooler during Hi-C matrix balancing and are ignored during visualization.

### PCA and linear model with feature selection

Values for all chromatin organization variables (see Fig. 4A, horizontal axis) were calculated for joint PRDM9–DSB sites, defined as the union of bins containing PRDM9 and DMC1-SSDS ChIP-seq peaks. Variables were preprocessed with standard scaling (zero, mean; one, standard deviation), followed by principal component analysis using the sklearn.decomposition (Pedregosa et al. 2011) package to extract loadings for each principal component. Principal components were calculated for all PRDM9 sites and supplied (alongside PRDM9 ChIP-seq score and chromosomal position) as predictors to a linear model predicting DMC1-SSDS ChIP-seq score using forward variable selection (Supplemental Fig. S5B). Briefly, the forward selection process begins with a null model and adds variables one-by-one by choosing the most statistically significant predictor at each step into an updated least squares model, implemented using statsmodels.OLS (Seabold and Perktold 2010). The selection process converges when none of the remaining predictors passes the statistical significance threshold, in this case *P* < 0.001 with Bonferroni correction (significance threshold = 0.001/*n*, for *n* variables). The selected predictors and their respective *t*-statistics are then reported. The process was repeated for DMC1-SSDS ChIP-seq sites in a model that used principal components alongside DMC1-SSDS ChIP-seq score and chromosomal position as predictor variables to a linear model predicting crossover likelihood score.

### Software availability

Code used for analysis and visualization are available in Supplemental Code and also at GitHub (https://github.com/xiaofanjin/meiosis-recombination-chromatin).

## Supplementary Material

Supplemental Material
