# Supplementary material for: Genome-wide variability in recombination activity is associated with meiotic chromatin organization
Source: Genome Res. 2021 Sep;31(9):1561–72. doi: 10.1101/gr.275358.121 (PMC8415379; doi:10.1101/gr.275358.121)
Supplement: Supplemental Material [file supp_31_9_1561__DC1.html]

Genome-wide variability in recombination activity is associated with meiotic chromatin organization — Supplemental Material 

# Genome-wide variability in recombination activity is associated with meiotic chromatin organization

## Supplemental Material

- Supplemental\_Material.pdf
- Supplemental\_Code.zip
