# Supplementary material for: Genome-wide variability in recombination activity is associated with meiotic chromatin organization
Source: Genome Res. 2021 Sep;31(9):1561–72. doi: 10.1101/gr.275358.121 (PMC8415379; doi:10.1101/gr.275358.121)
Supplement: Supplemental Material [file supp_gr.275358.121_Supplemental_Material_.pdf]

Supplemental Materials

S1 Datasets used in integrative analysis

The table below lists all datasets used in our integrative analysis. In choosing datasets to focus on when similar instances were available (e.g., meiotic Hi-C (Patel et al. 2019; Wang et al. 2019; Vara et al. 2019)), we seek where possible to maintain consistency in terms of mouse strain background. For instance, the PRDM9, DMC1 (DSB) and crossover datasets all analyzed recombination in a B6 x CAST hybrid context.

Data type	Cell/tissue type	Mouse strain information	Citation	Notes
Hi-C	E14TG2a Embryonic stem (ES) cell line	129/Ola background	(Bonev et al. 2017)	Used for ES Hi-C in Figs. 2-4.
Hi-C	Zygonema and pachynema spermatocytes	B6 x CAST	(Patel et al. 2019)	Used for zygonema, pachynema Hi-C in Figs. 1-4, mouse strain background matches PRDM9 and DSB datasets. (Baker et al. 2015; Smagulova et al. 2016)
Hi-C	Pachynema spermatocytes	B6 x PWK	(Wang et al. 2019)	Used for additional pachynema Hi-C in Fig. S4.
Hi-C	Lepto/zygonema, pachy/diplonema spermatocytes and round spermatids	B6	(Vara et al. 2019)	Used in Fig. S4.
Hi-C	Round spermatids	B6	(Alavattam et al. 2019)	Used in Fig. S4.

ChIP-seq (anti-CTCF, anti-REC8, anti-RAD21L)	Pachy/diplonema spermatocytes	B6	(Vara et al. 2019)	Used for meiotic ChIP-seq in Figs. 1-4.
ChIP-seq (anti-RNAPII)	Testis extract from 16 dpp mice	B6	(Margolin et al. 2014)	Used for meiotic ChIP-seq in Figs. 1-4.
ChIP-seq (anti-CTCF, RAD21)	ES-R1 ES cell line	129 background	(Nitzsche et al. 2011)	Used for ES ChIP-seq in Figs. 2-4.
ChIP-seq (anti-RNAPII)	Bruce4 ES cell line	B6 background	(ENCODE) (Shen et al. 2012)	Used for ES ChIP-seq in Figs. 2-4.
ChIP-seq (anti-PRDM9)	Whole tubule extract from 12 dpp mice	B6 x CAST	(Baker et al. 2015)	Used for PRDM9 binding signal in Figs. 1-4.
SSDS ChIP-seq (anti-DMC1)	Testis extract adult mice	B6 x CAST	(Smagulova et al. 2016)	Data used for double-strand-break (DSB) signal in Figs. 1-4.
Single-cell whole genome sequencing	Mature 1C sperm	B6 x CAST	(Yin et al. 2019)	Data used for crossover signal in Figs. 1-4.
ChIP-seq (anti-PRDM9)	Testis extract from 13 dpp mice	B6 and RJ2	(Grey et al. 2017)	RJ2 expresses PRMD9-CAST on C57BL/10 background. Used for PRDM9 CAST vs. B6 analysis in Fig. S1.

S2 Comparing B6 and CAST *Prdm9* allele results

PRDM9-related results presented in the main text are from ChIP-seq of a B6xCAST strain (Baker et al. 2015), which harbours two separate *Prdm9* alleles - the Dom2 allele from B6, and the CAST allele. To separately analyze chromatin conformation around B6 and CAST allele binding peaks, we use a different PRDM9 ChIP-seq dataset from Grey *et al.* which ran separate experiments for the B6 allele (on native B6 genetic background) and the CAST allele, on a very similar genetic background to B6 (RJ2 strain) (Grey et al. 2017). Unlike the Baker *et al.* dataset used in the main text, this dataset produced a number of binding peaks away from recombination hotspots (termed Class 2 binding, as opposed to Class 1 binding which refers to hotspots). Directly using peaks called in the original publication (Grey et al. 2017), ChIP-seq peaks were first classified as Class 1 and Class 2 by

filtering for intersections with DMC1 or H3K4me3 binding as described (Grey et al. 2017), and then lifted over from mm9 to mm10.

Comparing with the Baker *et al.* peaks, we observe strong overlap with Class 1 binding (Fig. S1A), but not Class 2 binding, indicating that non-hotspot binding largely did not occur with the Baker *et al.* dataset. We observe that Class 2 peaks have much lower levels of DSB and crossover enrichment compared to their Class 1 counterparts (Fig. S1B), and are strongly skewed to active A-compartment chromatin (Fig. S1C). This is also reflected in overall higher compartment scores and lower cis/total ratio at Class 2 peaks (Fig. S1D). We focus mainly hereafter on Class 1 peaks, which are more comparable to peaks from the Baker *et al.* dataset.

Comparing binding of the B6 and CAST *Prdm9* alleles, fewer peaks are detected with the native B6 allele (1881 Class 1 peaks) than the CAST allele (7218 Class 1) as reported in the original publication (Grey et al. 2017). Note mm9-mm10 lift-over process resulted in slight differences in counts from original publication, and that number of genomic sites (i.e., bins intersecting peaks) is less than the total number of peaks. In addition to fewer overall binding sites, B6 PRDM9 Class 1 sites tend to be less likely to form DSBs and crossovers as compared to CAST (Fig. S1B). Reduced B6 binding may be related to potential hotspot erosion (i.e., loss over evolutionary timescales of B6 PRDM9 protein binding sites on its native background) and reported dominance of CAST *Prdm9* allele over B6 (Grey et al. 2017; Smagulova et al. 2016).

Despite these differences, B6 PRDM9 Class 1 sites exhibit overall similar signals to CAST counterpart in terms of 3D organization measured by Hi-C (Figs. S1D). Both CAST and B6 sites exhibit local zygonema-specific shifts toward spatially accessible active configuration of low cis/total ratio and high compartment score. We also partition CAST and B6 PRDM9 Class 1 sites into the most and least DSB favored quartiles (Figs. S1E,F), revealing that consistent with conclusions from the main text, both CAST and B6 PRDM9 Class 1 sites are more likely to form DSBs if they are in active, spatially accessible chromatin environments. This suggests that these environments are associated with increased DSB formation in general, rather than specific to a particular PRDM9 allele.

S3 Haplotype resolved Hi-C analysis distinguishing B6-B6, CAST-CAST, and interhomolog reads

Pachynema and zygonema Hi-C datasets from Patel *et al.* were generated in a B6xCAST genotype. Given the 0.83% overall SNP density between B6 and CAST genotypes, it was possible to haplotype filter a majority of Hi-C contact reads based on strain (Patel et al. 2019). With haplotype filtered reads provided by the original authors (Patel et al. 2019), we generated contact matrices for unambiguous B6-B6 contacts, CAST-CAST contacts, and interhomolog contacts, and explored whether the cis/total ratio and compartment score patterns observed around PRDM9 sites were specific to a particular genetic background (Fig. S2A). We observe that key observations made using non-haplotype-filtered Hi-C hold true (compare to Fig. 2B), such as local, zygonema-specific increase in compartment score / decrease in cis/total ratio, and increased compartment score in DSB-favored sites. These

patterns also hold true using separate B6 and CAST allele analysis of PRDM9 binding (Figs. S2B,C), indicating the two different forms of the PRDM9 protein are acting on both their native and non-native chromosomes. Finally, we apply haplotype resolved analysis to observe cis/total ratio and compartment score patterns around DSB sites (Fig. S2D). Again patterns are similar to non-haplotype-resolved Hi-C (compare to Fig. 3B), with higher meiotic cis/total ratio associated with increased crossover likelihood. These findings suggest overall that genome folding patterns around PRDM9 sites are a general phenomenon not specific to a particular genetic background.

S4 Chromosomal organization around cohesin occupancy sites

Cohesin is involved in the assembly of chromosomal axes during meiosis, and localizes at the axial core of the brush-loop structure during meiosis (Llano et al. 2012; Suja and Barbero 2009; Vara et al. 2019; Lee and Hirano 2011; Ishiguro et al. 2011). We analyze the ChIP-seq data for the meiosis-specific cohesin subunit RAD21L in pachynema-diplonema spermatocytes (Vara et al. 2019), as well as of its interphase counterpart RAD21 in ES cells (Nitzsche et al. 2011). We also consider REC8, another meiotic-specific cohesin kleisin subunit. Earlier microscopy results indicate that spatial occupancy patterns of RAD21L and REC8 are mutually exclusive on individual chromosomes (Lee and Hirano 2011; Ishiguro et al. 2011). Our analysis based on bulk ChIP-seq data, however, indicates that at 5kb bin resolution, REC8 and RAD21L sites often overlap (Fig. S3A), consistent with original analysis of the dataset (Vara et al. 2019). Follow-up work could explore whether there exists a universal occupancy pattern dictating mutual exclusion of REC8 versus RAD21L occupancy, or whether cell-to-cell differences in occupancy patterns dominate. By comparison, meiotic RAD21L and REC8 cohesin sites overlap much less frequently with interphase RAD21 sites. While all three cohesin subunits have occupancy skewed toward A-compartment chromatin, this effect is most pronounced with meiotic subunits (Fig. S3A).

Given that cohesin is expected to typically converge at the base of chromatin loops (e.g. along chromosomal axes during meiosis) with low spatial accessibility, we expect sites of cohesin occupancy to exhibit high cis/total ratio. While this is the case for the interphase RAD21 subunit measured in ES cells, we find that meiosis-specific cohesin sites instead exhibit low cis/total ratios (Fig. S3B). This puzzling observation also contrasts with those for yeast meiosis, where Rec8 cohesin sites are associated with local maxima in the cis/total ratio (Schalbetter et al. 2019).

Digging deeper, the differences in average spatial accessibility across cohesin types during pachynema (Patel et al. 2019) appear related to the enrichment of RAD21L sites at active promoter-like regions marked by H3K4me3 (Fig. S3C) and away from unmarked (i.e., generally more quiescent) chromatin regions. Splitting the cohesin sites by ChromHMM group prior to averaging, we observe that cis/total ratio trends are more similar across different cohesin subunits, in particular the meiotic subunits REC8 and RAD21L (Fig. S3D). Indeed, elevated cis/total ratio patterns are observed at RAD21L sites not associated with H3K4me3 or H3K36me3 marks typical of active promoters and gene bodies (Fig. S3D), much like interphase RAD21. This suggests that decreased

cis/total ratio at meiotic RAD21L sites may be associated with RAD21L's increased colocalization with active promoter/enhancer-like histone marks and transcriptional activity relative to interphase RAD21. RAD21L sites also exhibit a modest enrichment for H3K27me3 marks (Fig. S3C) typical of repressed promoter regions, suggesting that enrichment of meiotic cohesin towards promoters may occur irrespective of active-vs.-repressed promoter state. H3K27me3-marked RAD21L sites are characterized by increased cis/total ratio (Fig. S3D).

Other explanations for low meiotic cis/total at RAD21L sites can include technical aspects of dataset collection. In particular, the RAD21L ChIP-seq dataset was generated from pachynema/diplonema spermatocytes (Vara et al. 2019), representing a later stage of meiotic prophase compared to the zygonema and pachynema Hi-C datasets from Patel *et al.* (Patel et al. 2019). Therefore, it is possible that stable cohesin accumulation at RAD21L ChIP-seq sites is present in later stage pachynema/diplonema spermatocytes, but not in zygonema or early pachynema. Indeed, previous work suggests that ongoing loop extrusion occurs during earlier stages of meiotic prophase I (Patel et al. 2019). Consistent with loop extrusion continuing until late prophase, we detect modest local maxima for the cis/total ratio at RAD21L sites in post-meiotic round spermatids, pachynema/diplonema stage spermatocytes, as compared with low cis/total ratio in early prophase (Fig. S3E). We find a similar modest local elevation in another recently published pachynema Hi-C dataset published by Wang *et al.* (Wang et al. 2019) (Fig. S3B). We speculate this dataset may capture spermatocytes at a slightly later stage of pachynema than that of Patel *et al.*

Motivated by Hi-C patterns around cohesin sites during interphase, we next test for evidence of boundary demarcation at meiotic cohesin sites and enriched contacts between site pairs by piling up Hi-C maps averaged around these features. We observe evidence of both boundary demarcation at individual cohesin sites during meiosis, as reported by Vara *et al.* (Vara et al. 2019), as well as enriched Hi-C contacts between proximal cohesin site pairs for RAD21, RAD21L and REC8 sites (Fig. S3F). These signals are more evident in pachynema than zygonema, especially for meiosis-specific subunit RAD21L, though overall much weaker than analogous RAD21-based signals in ES cells (i.e., interphase). Similar patterns are also observed in the pachynema Hi-C dataset by Wang *et al.* (Fig. S3F) (Wang et al. 2019). Given the REC8/RAD21L site overlap, averaged Hi-C signals at REC8 sites are similar to those of RAD21L (Fig. S3F).

Since previous work reported a relationship between cohesin positioning and transcriptional direction in yeast (Sun et al. 2015), and we observed enrichment of meiotic cohesin occupancy around promoter regions (Fig. S3C), we next explored whether transcriptional direction plays a role in genome folding patterns. By averaging meiotic Hi-C maps at RAD21L sites that uniquely intersect either positive or negative strand transcription start sites (TSS), we find that boundary signals occur both upstream and downstream regardless of strandedness, though downstream Hi-C contact frequency is modestly enriched for RAD21L sites intersecting +strand TSS and vice versa (Fig. S3G). This signal may be due to presence of enriched Hi-C contacts associated with gene bodies downstream of TSS. No clear differences are observed between convergent versus divergent RAD21L site pairs, which both show enrichment (Fig. S3G) – thus we are unable to clearly conclude a link between transcriptional direction and cohesin occupancy.

In addition to weak boundaries in Hi-C contact maps, we also observe minima in the log-insulation score (Fig.

S3B) at RAD21L sites, as expected given the boundary signals described above. Insulation minima are observed in both meiotic and interphase (ES) Hi-C. This opens the possibility that these loci are partially predetermined by cohesin occupancy prior to meiotic entry, though sample contamination by different cell types or meiotic stages cannot be ruled out. Meanwhile, ES RAD21 cohesin site loci exhibit very strong boundary demarcation and dot-loop-contact signals in matching ES Hi-C, but a much weaker signal in meiotic Hi-C (Fig. S3F).

Overall, these observations indicate that pachynema/diplonema stage cohesin ChIP-seq sites, particularly for meiosis-specific RAD21L, *should not* be directly interpreted as stable locations of cohesin occupancy – and by extension the brush-loop axis – throughout meiosis. Cis/total ratios at cohesin sites exhibit minima during early prophase, and appear to increase during later prophase as well as in post-meiotic Hi-C (Figs. S3B,E). This suggests a potential configuration where these promoter and CTCF-enriched genomic positions increasingly arrive at the cohesin-anchored axis during meiosis, perhaps due to an increasing tendency for these loci to stall cohesin extrusion. Noting that the chromatin boundary and loop-enrichment signals at RAD21L sites / site pairs are more apparent during pachynema compared to zygonema (Fig. S3F), we speculate that these ChIP-seq sites may be characterized by increasing cohesin accumulation – and by extension axial localization – as meiotic prophase I progresses.

S5 Cis/total ratio of DSB sites split by ChromHMM state

In the main text, we discuss how selection of crossovers from DSBs is depleted in genomic regions with low cis/total ratio (i.e., high spatial accessibility) particularly as measured from meiotic Hi-C, as well as in regions with H3K36me3 histone annotation from the mouse testis ChromHMM dataset. Here we explore the relationship between these two observations - specifically, we seek to address whether DSB sites in H3K36me3 chromatin are inherently characterized by low cis/total ratio, and if so whether cis/total ratio is a relevant factor for crossover selection in ChromHMM annotated regions beyond H3K36me3 regions. To investigate this, we first split DSB sites by their ChromHMM annotation thus forming seven groups. For each individual group, we partitioned by crossover likelihood score, and plot the averaged pachynema cis/total ratio for the top and bottom quartile DSB sites of each group (Fig. S4). We note that H3K36me3 associated DSB sites are indeed characterized by minima in cis/total ratio. However, we also note that the tendency of low cis/total ratio sites to be depleted for crossover selection is observed in all ChromHMM groups, not just H3K36me3-associated. Thus it appears that high spatial accessibility of chromatin during meiosis is associated with depleted crossover formation in general.

S6 Collinearity between variables describing chromatin organization and principal component analysis

A total of 50 variables (see x-axis of Fig. 4A) describe chromatin organization at joint PRDM9-DSB sites (i.e., the union of PRDM9 sites and DSB sites). To deal with collinearity between these variables (Fig. S5A), PCA is

performed across these sites, transforming the 50 variables into a 50-dimensional principal component space (Fig. S5B). These transformed principal components (PCs), along with chromosomal position and PRDM9 ChIP-seq score, serve as inputs to the first linear model with forward selection to predict DMC1-SSDS ChIP-seq score (i.e., DSB score). The second linear model predicts crossover score, using DMC1-SSDS ChIP-seq score in place of PRDM9 ChIP-seq score. (Fig. S5B) PCs are ordered by percent explained variance (Fig. S5C) of each component.

S7 Partitioning PRDM9 sites by crossover score

In addition to separately assessing two stages of recombination (PRDM9-to-DSB in Fig. 2 and DSB-to-crossover in Fig. 3), we also apply our approach to analyze PRDM9 sites partitioned based on their crossover score (i.e., PRDM9-to-crossover). This analysis indicates that PRDM9 sites least favored for crossover formation (bottom-CO) still have elevated PRDM9 and DMC1 binding, though less than their top-CO counterparts – on the other hand, they exhibit very low levels of crossover formation (Fig. S6A). Crossover formation appears most favored in sites with high meiotic cis/total ratio, as well as high compartment scores (Fig. S6B), i.e. active chromatin with low spatial accessibility. This is an interesting combination, as high compartment scores typically correlate with low cis/total ratio (Fig. S5A), and appears to reflect opposing chromatin constraints for the PRDM9-to-DSB and DSB-to-crossover stages of recombination. PRDM9 sites in gene bodies, though capable of forming DSBs (Fig. 2F), are rarely selected for eventual crossovers (Fig. S6C). Results with PCA and linear model analysis indicate that chromosomal position and PRDM9 ChIP-seq score positively effect crossover score, suggesting PRDM9 peaks with natural affinity for PRDM9 binding are more favored to form crossovers, and crossovers are depleted near centromeres. In addition, PC2 and PC3 both show significant negative effect on crossover formation, indicating that PRDM9 sites in gene bodies and regions with high spatial accessibility are disfavored for crossovers.

S8 Correlation between crossovers and gene bodies

Earlier work has indicated that at genome-wide scale, positive correlations exist between presence of gene bodies and crossover formation (Yin et al. 2019). While this may initially seem to differ from our conclusions that crossovers are depleted in gene bodies, we show here that this apparent discrepancy is due to our analysis focusing on genomic bins corresponding to joint PRDM9-DSB sites (rather than all bins genome-wide). At global, genome-wide scale, both crossovers and gene-bodies are enriched in A-compartment, and accordingly a positive correlation between the two exist considering all 5kb bins in the genome (Fig. S7A). However, a more localized analysis calculating correlation only joint PRDM9-DSB sites reveals a negative correlation (Fig. S7B), in line with our findings of crossover depletion in gene bodies.

S9 Differences in loop lengths between A and B-compartment

Earlier cytological observations of meiotic chromosomes indicated two findings: (i) loop densities are relatively constant along chromosomal axes (Zickler and Kleckner 1999), and (ii) euchromatin / R-band chromatin – roughly corresponding to A-compartment – is over-represented (i.e., stretched) along the physical length of the axis (Luciani et al. 1988; Fransz et al. 2000). By contrast, this effect is not observed in mitotic chromatin loop arrays. This suggests that for the same genomic distance, A-compartment chromatin forms more loops than B-compartment chromatin, implying that loops in A-compartment regions should have fewer base-pairs than B-compartment. To explore whether such a configuration can be observed in meiotic Hi-C datasets, we apply the $P(s)$ derivative method for estimating loop lengths (Gassler et al. 2017; Patel et al. 2019). Briefly, by calculating the derivative of the contact frequency versus distance curve – commonly referred to as $P(s)$ curve – the genomic distance where the derivative equals zero can be used as an estimate of loop length.

We apply this technique to meiotic Hi-C contact matrices masking A-compartment and B-compartment regions respectively, and find indeed that there exists an approximately 2-3 fold difference in the number of base pairs in A versus B-compartment loops in zygonema (Fig. 5A), with the local maxima of the $P(s)$ curve shifted to the right. This finding is replicated in multiple pachynema Hi-C datasets (Figs. S8A-C). Notably, we do not observe the same patterns in a mitotic Hi-C dataset (Gibcus et al. 2018) (Fig. S8D). We do observe similar patterns in a DSB-deficient TOP6BL-/- Hi-C dataset (Wang et al. 2019) (Fig. S8E,F), suggesting that shorter loops in A-compartment are not primarily caused by increased loop tethering due to DSB formation in A-compartment DNA.

S10 Interhomolog Hi-C contacts are reduced in gene bodies and elevated at crossover-favored DSBs.

We investigate the rates of interhomolog contacts in different chromatin regions and the effects on crossover formation. To account for SNP density differences between different genomic regions, we normalize interhomolog Hi-C contacts, defined as contacts unambiguously mapped as B6-CAST, by intrahomolog contacts unambiguously mapped as B6-B6 or CAST-CAST. This normalized inter-vs.-intrahomolog contact frequency is lower in gene bodies (Fig. S9A) and higher in crossover favored DSBs (Fig. S9B). This suggests that decreased crossover formation at DSBs in gene bodies may be related to a lower level of stable, interhomolog contacts in gene bodies. Differences are not observed between top vs. bottom DSB forming PRDM9 sites (Fig. S9B).

S11 Additional details on generating genetic map of crossover scores from single-sperm-seq dataset

Single sperm sequencing data from Yin *et al.* (Yin et al. 2019) provides a list of chromosomal positions (i.e., chrom, start-bp, end-bp) of crossover loci. Some loci are sharply localized, meaning the end-bp is close to the start-bp, while others are less well localized and span a greater genomic range. The coverage of each of these positions is mapped to a set of 5kb bins across the genomic, inversely weighted by the length of each crossover (i.e. end-bp minus start-bp), to give prominence to sharply localized crossovers. This can be visualized as each crossover occupying a 2D rectangle above the genomic axis, with width corresponding to the start-bp and end-bp. All crossovers occupy an equal area rectangle, and the overall crossover score is obtained by stacking these rectangles on top of each other and tracing the upper boundary (Fig. S10).

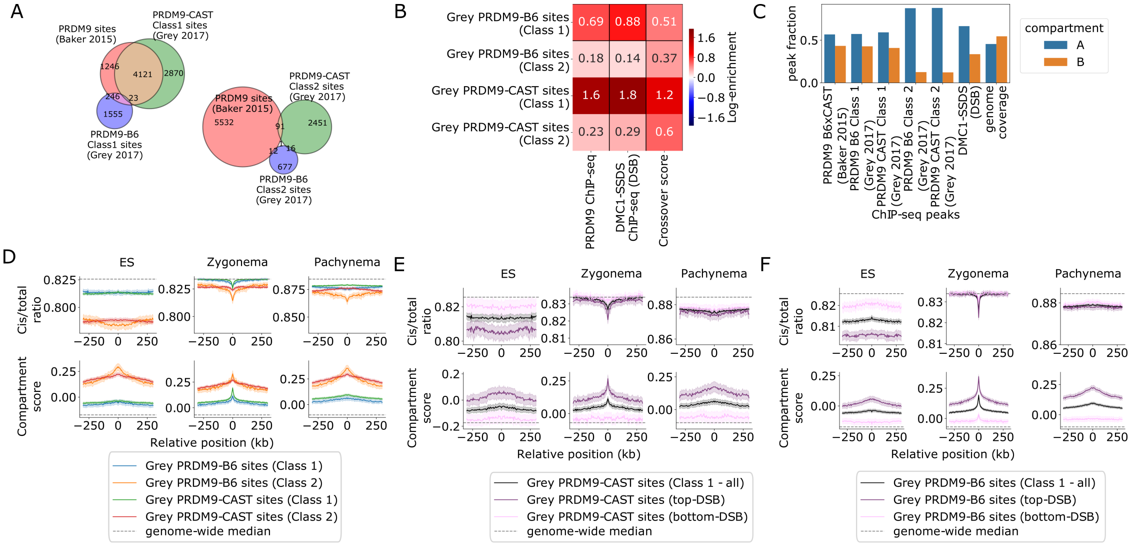

Figure S1: Comparison between binding sites of CAST and B6 *Prdm9* alleles. **A:** Overlap between B6xCAST PRDM9 sites used in main text (from Baker *et al.*) with PRDM9-CAST and PRDM9-B6 sites from Grey *et al.*. Note significant overlap with Class 1 sites, but not with Class 2, which are largely absent in Baker *et al.* dataset. **B:** PRDM9, DMC1 and crossover enrichment at Class 1 and Class 2 PRDM9 sites for B6 and CAST alleles. Heatmap shows log fold enrichment over genome median. Note Class 2 sites exhibit much lower recombination activity. **C:** Fraction of sites that intersect A-active vs B-inactive compartment, for B6xCAST PRDM9 (Baker *et al.*), PRDM9 B6 Class 1/2 and CAST Class 1/2 sites (Grey *et al.*), as well as DSB sites (B6xCAST, Smagulova *et al.*). Compartments defined using ES Hi-C dataset, total genomic fraction of A-B bins shown for reference. Note DSB sites are more A-compartment skewed than PRDM9 B6xCAST sites, consistent with DSB formation bias in active chromatin. Class 2 PRDM9 sites are strongly A-compartment skewed. **D:** Hi-C cis/total ratio (top), and compartment score (bottom) averaged across all PRDM9 Class 1 and 2 sites for B6 and CAST alleles. **E:** Hi-C cis/total ratio (top), and compartment score (bottom) averaged across PRDM9-CAST Class 1 sites, partitioned by DSB score. Compare with Fig. 2B in main text. **F:** Hi-C cis/total ratio (top), and compartment score (bottom) averaged across PRDM9-B6 Class 1 sites, partitioned by DSB score. Compare with Fig. 2B in main text.

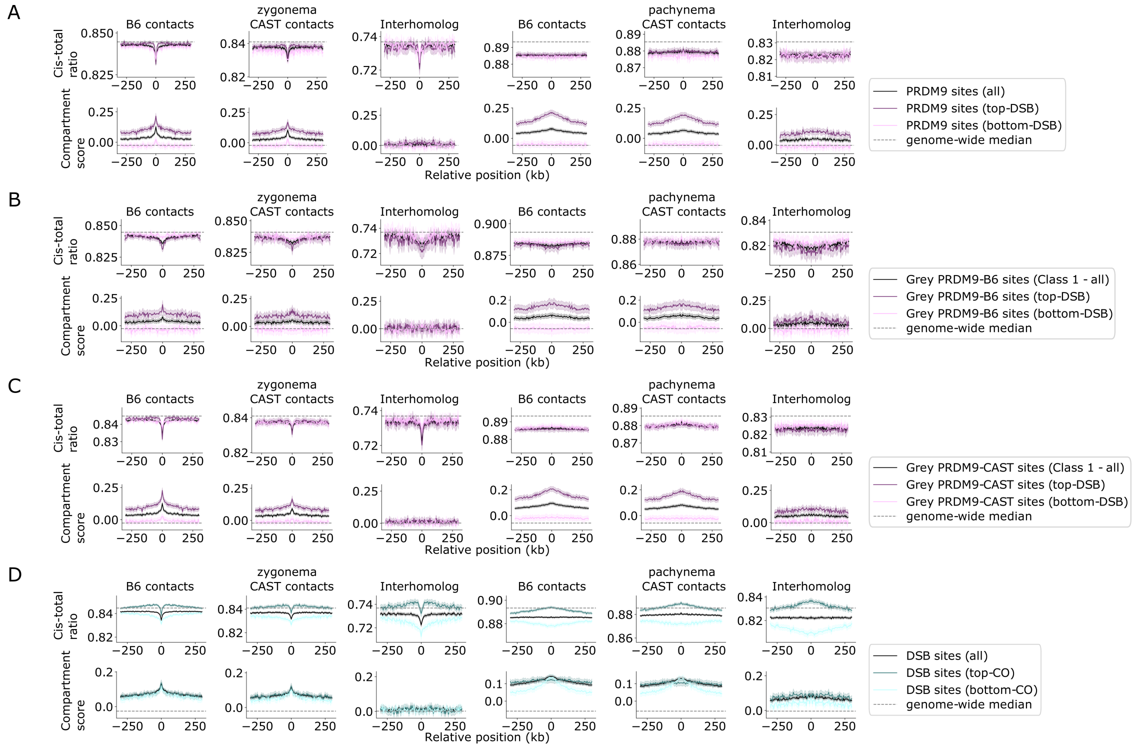

Figure S2: Cis/total ratio and compartment score analysis of PRDM9 and DSB sites using haplotype resolved Hi-C. **A:** Haplotype resolved Hi-C cis/total ratio (top), and compartment score (bottom), for zygonema (left) and pachynema (right), averaged across PRDM9 B6xCAST sites (from Baker *et al.*), partitioned by DSB score. B6 contacts indicates data generated from Hi-C contacts unambiguously assigned as B6-B6, CAST contacts indicates data generated from Hi-C contacts unambiguously assigned as CAST-CAST, and interhomolog indicates data generated from Hi-C contacts unambiguously assigned as CAST-B6. **B:** Haplotype resolved Hi-C cis/total ratio (top), and compartment score (bottom), for zygonema (left) and pachynema (right), averaged across PRDM9-B6 sites (from Grey *et al.*), partitioned by DSB score. **C:** Haplotype resolved Hi-C cis/total ratio (top), and compartment score (bottom), for zygonema (left) and pachynema (right), averaged across PRDM9-CAST sites (from Grey *et al.*), partitioned by DSB score. **D:** Haplotype resolved Hi-C cis/total ratio (top), and compartment score (bottom), for zygonema (left) and pachynema (right), averaged across DSB sites, partitioned by crossover score.

Figure S3 *(following page)*: ChIP-seq occupancy patterns for cohesin subunits in relation chromosomal organization. **A:** Overlap in occupancy sites for two meiotic cohesin subunits REC8 and RAD21L, and interphase RAD21 cohesin subunit from ES cells (top-left). Comparison of RNAPII, CTCF and cohesin occupancy overlaps in meiosis (bottom-left) and ES (top-right). Fraction of sites that intersect A-active vs B-inactive compartment, for cohesin, CTCF and RNAPII in ES and meiosis. Compartments defined using ES Hi-C dataset, total genomic fraction of A-B bins shown for reference (bottom-right). **B:** Hi-C cis/total ratio (top), compartment score (middle) and log insulation score (bottom), averaged across RAD21, REC8 and RAD21L sites, calculated for ES, zygonema, and two pachynema datasets. Shading represents 95% confidence intervals. Meiotic REC8 and RAD21L sites are associated with clear dips in the log-insulation score across meiotic and ES datasets – dips are largely confined to the ES dataset for RAD21 sites. All cohesin sites are associated with positive A-B compartment scores, especially RAD21L. Meiotic RAD21L sites are generally associated with minima in cis/total, whereas the opposite effect is observed at RAD21 sites. However, in the Wang *et al.* pachynema dataset, a small but distinct positive cis/total ratio spike (black arrow) can be observed at RAD21L and REC8 sites. **C:** Overlap of ChromHMM histone annotations with meiotic REC8, RAD21L and ES RAD21 sites. RAD21L exhibits strongest enrichment towards promoter-like histone marks (H3K4me3/H3K27me3, i.e. both active and inactive), and away from unmarked chromatin. Heatmap shows log fold enrichment over genome-wide mean, Insets plot overlap fraction averaged around cohesin sites, shading represents 95% confidence intervals. **D:** Cis/total ratio derived from pachynema Hi-C (Patel et al. 2019), averaged across cohesin binding sites split by ChromHMM epigenetic category. Note that meiotic cohesin sites not intersecting active promoters or gene bodies (H3K4me3 / H3K36me3) display patterns of cis/total ratio maxima. **E:** Cis/total ratio averaged across RAD21, RAD21L and REC8 sites, derived from additional Hi-C datasets: lepto/zygonema, pachynema/diplonema spermatocytes (Vara et al. 2019) and round spermatids (Vara et al. 2019; Alavattam et al. 2019). Note elevated cis/total ratio at cohesin sites in these later meiotic / post-meiotic datasets datasets. **F:** (Top left) Schematic of Hi-C window averaging approach, with orange on-diagonal windows at ChIP-seq sites, and purple windows at off-diagonal site-pairs. Normalized chromatin contact matrices, averaged across meiotic RAD21L (bottom left), REC8 (top right) and ES RAD21 (bottom right) sites (orange) / bin-pairs (purple), for ES, zygonema and pachynema Hi-C datasets introduced in the main text from Bonev *et al.* / Patel *et al.* (Bonev et al. 2017; Patel et al. 2019), and an additional pachynema dataset from Wang *et al.* (Wang et al. 2019). During meiosis and in ES cells, RAD21L ChIP-seq sites exhibit boundary-like contact patterns, and site pairs exhibit enriched Hi-C contacts – patterns become more evident as meiosis progresses from zygonema to pachynema. RAD21 sites exhibit similar contact patterns strongly in the ES interphase Hi-C dataset, but weakly during meiosis (note different color-scale). **G:** (Left) Averaged pachynema (Patel et al. 2019) Hi-C contact maps for RAD21L sites that uniquely intersect negative and positive strand transcription start sites (TSS). Downstream and upstream contacts are enriched for + and - strand sites respectively. (Right) Averaged pachynema (Patel et al. 2019) Hi-C contact maps for RAD21L bin-pairs with convergent and divergent TSS.

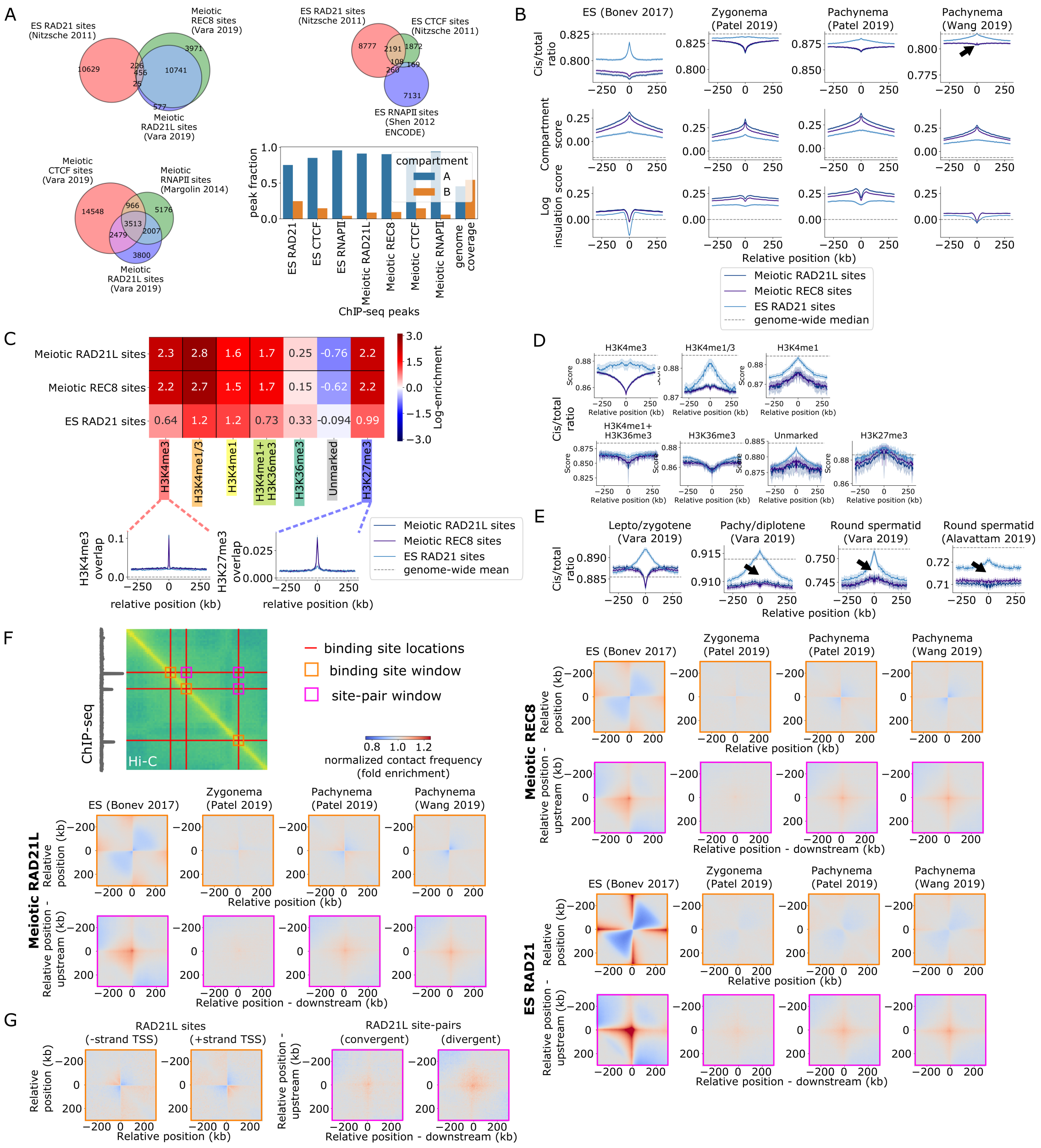

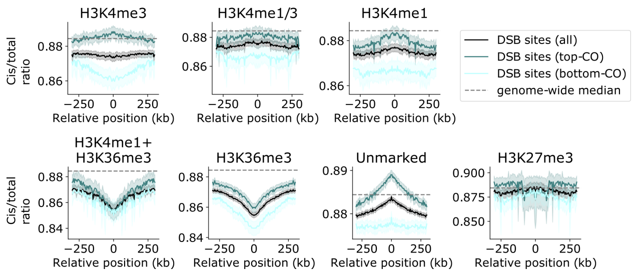

Figure S4: Pachynema cis/total ratio traces averaged across DSB sites. DSB sites were first split by ChromHMM annotation into seven categories, then for each category partitioned by crossover score into top and bottom quartiles.

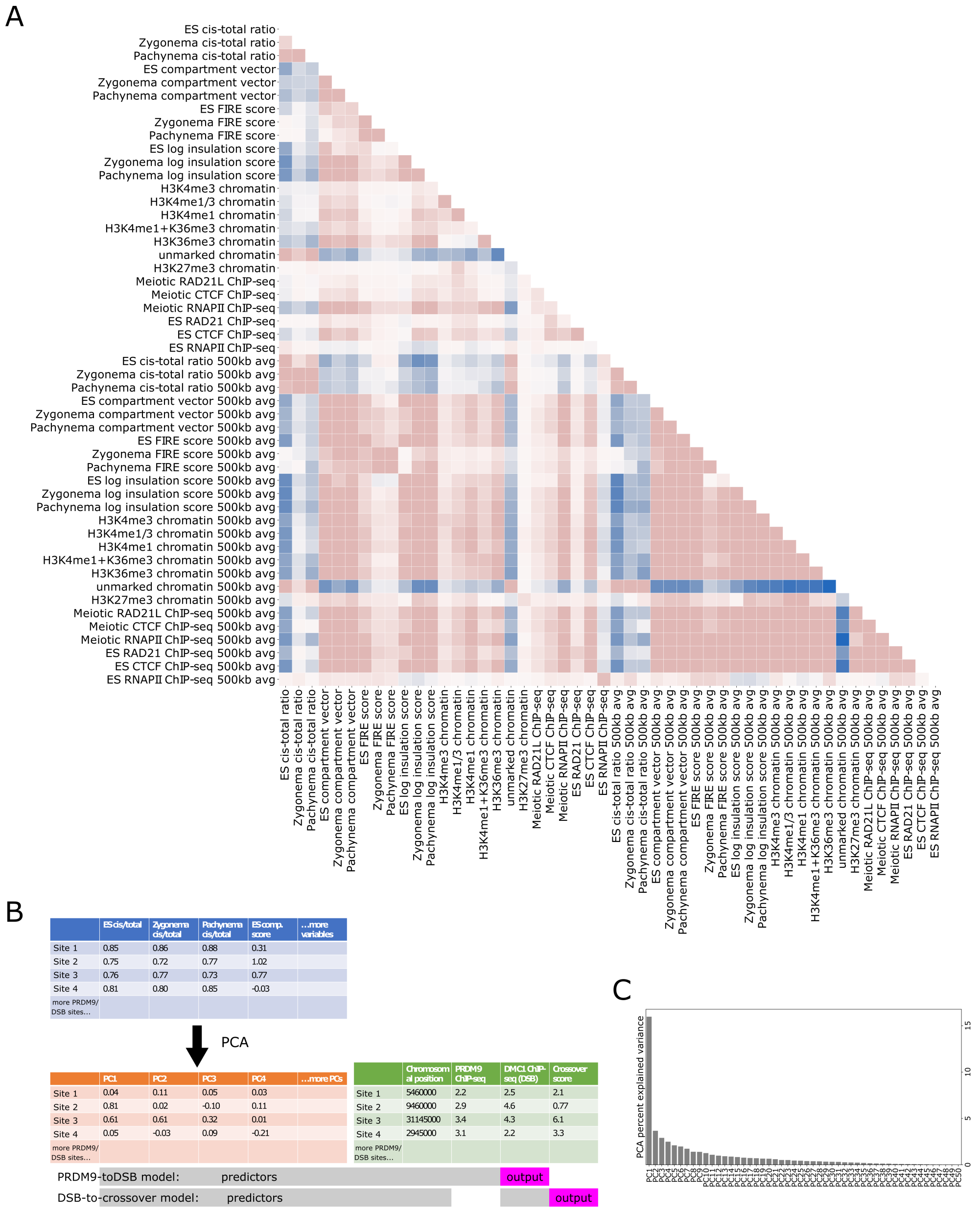

Figure S5: Application of principal component transformation to address collinearity between chromatin organization variables **A:** Collinearity between chromatin organization variables is demonstrated by plotting pairwise Spearman correlation coefficients between all variables. **B:** Schematic depicting how principal component analysis is applied on chromatin organization variables to generate PCs, which are then used as input predictors along with chromosomal position and intrinsic ChIP-seq score (marked in grey) to linear models predicting DSB and crossover formation (predicted outputs in purple). Note that values and PRDM/DSB sites are made-up examples. **C:** Percent explained variance for each principal component.

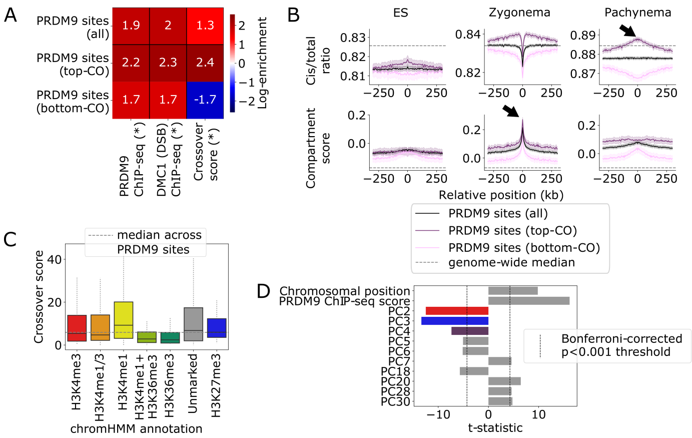

Figure S6: PRDM9-to-crossover partitioning indicates crossovers favored at PRDM9 sites in active, spatially inaccessible chromatin. **A:** Summary of recombination activity at PRDM9 sites, partitioned into the top and bottom quartiles of crossover score. Bottom-CO (i.e., CO-disfavored) sites only exhibit slightly less strong PRDM9 binding and DSB formation, in comparison to strong crossover depletion. Heatmap shows log fold enrichment over genome median, while (*) indicates Bonferroni-adjusted p<0.01 difference between top and bottom partitioned sites. **B:** Hi-C cis/total ratio (top) and compartment score (bottom), averaged across CO-partitioned PRDM9 sites, calculated for ES, zygonema and pachynema datasets. Shading represents 95% confidence intervals. Top CO-favored sites are associated with higher compartment score as well as higher cis/total ratio during meiosis (black arrows). **C:** Distribution of crossover scores at PRDM9 sites split by ChromHMM state. Crossover formation is depleted within H3K36me3-marked chromatin characteristic of gene bodies. **D:** Results from linear model with feature selection, predicting crossover score at PRDM9 sites.

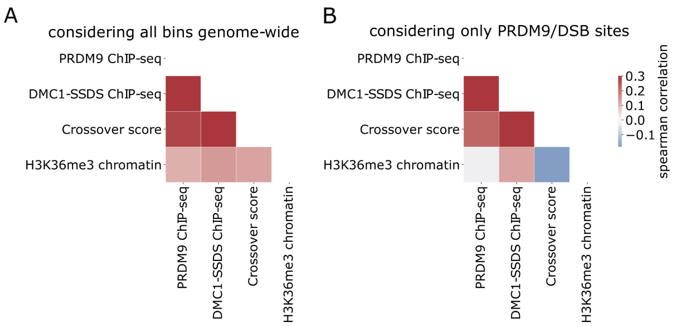

Figure S7: Correlation between crossover score and H3K36me3 is positive when calculated genome-wide, but negative considering only PRDM9 and DSB sites. **A:** Correlation between H3K36me3 ChromHMM overlap and recombination variables calculated for all 5kb bins genome-wide. **B:** Correlation between H3K36me3 ChromHMM overlap and recombination variables calculated for 5kb bins at intersect either a PRDM9 or DMC1-SSDS ChIP-seq peak.

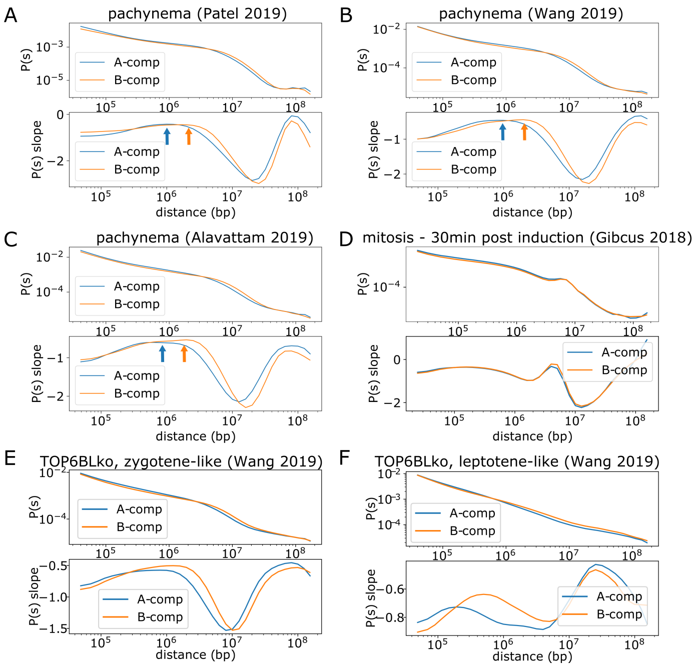

Figure S8: A-compartment loops have fewer base pairs, meaning active chromatin is closer to the chromosomal axis on average in terms of genomic distance. **A:** $P(s)$ curve derivative analysis applied to pachynema dataset from Patel *et al.* (Patel et al. 2019). Orange and blue arrows indicate estimate of loop length for A and B compartments respectively, determined as the maxima of the derivatives of the $P(s)$ curves as in Gassler *et al.* (Gassler et al. 2017). **B:** $P(s)$ curve derivative analysis applied to pachynema dataset from Wang *et al.* (Wang et al. 2019) **C:** $P(s)$ curve derivative analysis applied to pachynema dataset from Alavattam *et al.* (Alavattam et al. 2019) **D:** $P(s)$ curve derivative analysis applied to mitotic dataset from Gibcus *et al.* (Gibcus et al. 2018). Note A and B compartment curves nearly overlap, unlike in meiosis where B-compartment curves are clearly shifted to the right. **E:** $P(s)$ curve derivative analysis applied to TOP6BL-/-, zygonema-like dataset from Wang *et al.* (Wang et al. 2019) **F:** $P(s)$ curve derivative analysis applied to TOP6BL-/-, leptonema-like dataset from Wang *et al.* (Wang et al. 2019). Note this dataset lacks features found in other meiotic Hi-C datasets, such as contact frequency drop-off around 10Mb.

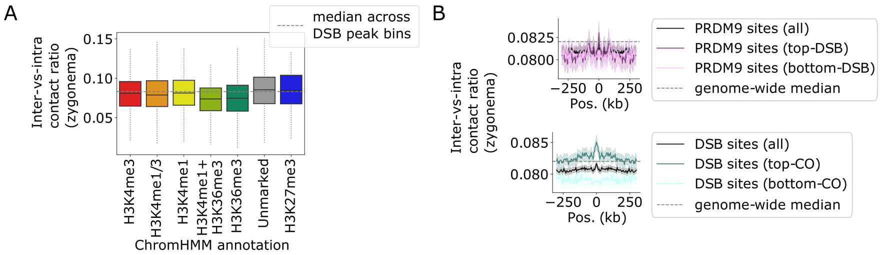

Figure S9: Interhomolog (B6-CAST) Hi-C contact frequency normalized by intrahomolog (B6-B6 + CAST-CAST) contact frequency is lower in gene bodies and higher in crossover favored DSBs. **A:** Interhomolog vs. intrahomolog contact frequency at DSBs classified by ChromHMM state. Interhomolog contact ratio (from zygonema Hi-C) is lowest in H3K36me3 gene body regions, which are also characterized by low crossover rates (Fig. S3F). **B:** Interhomolog vs. intrahomolog contact frequency, symmetric-averaged across DSB sites. Top crossover DSB sites are characterized by higher interhomolog contact frequencies, while differences are not observed between top vs. bottom DSB forming PRDM9 sites.

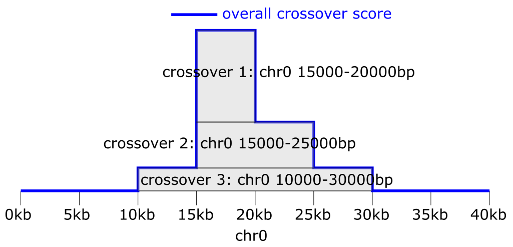

Figure S10: Visual representation of method to generate crossover scores. Toy example for an imaginary chromosome named chr0, onto which three crossover tracts are mapped at 5kb bin resolution. Crossover 1 has start-bp 15000, end-bp 20000, crossover 2 has start-bp 15000, end-bp 25000, and crossover 3 has start-bp 10000, end-bp 30000. Overall crossover score for a given bin is obtained by summing contributions of all crossovers intersecting the bin, with each crossover's contribution inversely weighted by length of the crossover tract (i.e., each of 3 grey boxes depicting crossovers has an equal area). Note in reality, start-bp and end-bp will not perfectly line up with bin widths, contribution of partial overlaps to a bin are weighted by the overlap fraction (e.g., a crossover tract that intersects 2500bp of a bin will have it's contribution halved for that bin).

References

Alavattam KG, Maezawa S, Sakashita A, Khoury H, Barski A, Kaplan N, and Namekawa SH. 2019. Attenuated chromatin compartmentalization in meiosis and its maturation in sperm development. *Nature Structural and Molecular Biology*. **26**: 175–184.

Baker CL, Kajita S, Walker M, Saxl RL, Raghupathy N, Choi K, Petkov PM, and Paigen K. 2015. PRDM9 Drives Evolutionary Erosion of Hotspots in Mus musculus through Haplotype-Specific Initiation of Meiotic Recombination. *PLOS Genetics*. **11**: e1004916.

Bonev B et al. 2017. Multiscale 3D Genome Rewiring during Mouse Neural Development. *Cell*. **171**: 557–572.e24.

Fransz PF, Armstrong S, Jong J de, Parnell LD, Drunen C van, Dean C, Zabel P, Bisseling T, and Jones GH. 2000. Integrated Cytogenetic Map of Chromosome Arm 4S of A.thaliana: Structural Organization of Heterochromatic Knob and Centromere Region. *Cell*. **100**: 367–376.

Gassler J, Brandão HB, Imakaev M, Flyamer IM, Ladstätter S, Bickmore WA, Peters JM, Mirny LA, and Tachibana K. 2017. A mechanism of cohesin-dependent loop extrusion organizes zygotic genome architecture. *The EMBO journal*. **36**: 3600–3618.

Gibcus JH et al. 2018. A pathway for mitotic chromosome formation. *Science*. **359**:

Grey C, Clément JAJ, Buard J, Leblanc B, Gut I, Gut M, Duret L, and Massy B de. 2017. In vivo binding of PRDM9 reveals interactions with noncanonical genomic sites. *Genome research*. **27**: 580–590.

Ishiguro Ki, Kim J, Fujiyama-Nakamura S, Kato S, and Watanabe Y. 2011. A new meiosis-specific cohesin complex implicated in the cohesin code for homologous pairing. *EMBO reports*. **12**: 267–275.

Lee J and Hirano T. 2011. RAD21L, a novel cohesin subunit implicated in linking homologous chromosomes in mammalian meiosis. *Journal of Cell Biology*. **192**: 263–276.

Llano E, Herrán Y, García-Tuñón I, Gutiérrez-Caballero C, Álava E de, Barbero JL, Schimenti J, Rooij DG de, Sánchez-Martín M, and Pendás AM. 2012. Meiotic cohesin complexes are essential for the formation of the axial element in mice. *The Journal of cell biology*. **197**: 877–885.

Luciani JM, Guichaoua MR, Cau P, Devictor B, and Salagnon N. 1988. Differential elongation of autosomal pachytene bivalents related to their DNA content in human spermatocytes. *Chromosoma*. **97**: 19–25.

Margolin G, Khil PP, Kim J, Bellani MA, and Camerini-Otero RD. 2014. Integrated transcriptome analysis of mouse spermatogenesis. *BMC genomics*. **15**: 39.

Nitzsche A et al. 2011. RAD21 Cooperates with Pluripotency Transcription Factors in the Maintenance of Embryonic Stem Cell Identity. *PLOS ONE*. **6**: e19470.

Patel L, Kang R, Rosenberg SC, Qiu Y, Raviram R, Chee S, Hu R, Ren B, Cole F, and Corbett KD. 2019. Dynamic reorganization of the genome shapes the recombination landscape in meiotic prophase. *Nature Structural and Molecular Biology*. **26**: 164–174.

Schalbetter SA, Fudenberg G, Baxter J, Pollard KS, and Neale MJ. 2019. Principles of meiotic chromosome assembly revealed in S. cerevisiae. *Nature Communications*. **10**: 4795.

Shen Y et al. 2012. A map of the cis-regulatory sequences in the mouse genome. *Nature*. **488**: 116–120.

Smagulova F, Brick K, Pu Y, Camerini-Otero RD, and Petukhova GV. 2016. The evolutionary turnover of recombination hot spots contributes to speciation in mice. *Genes & development*. **30**: 266–280.

Suja J and Barbero J 2009. Cohesin Complexes and Sister Chromatid Cohesion in Mammalian Meiosis. In: *Genome Dynamics*. Vol. 5. Karger, pp. 94–116.

Sun X, Huang L, Markowitz TE, Blitzblau HG, Chen D, Klein F, and Hochwagen A. 2015. Transcription dynamically patterns the meiotic chromosome-axis interface. *eLife*. **4**: e07424.

Vara C et al. 2019. Three-Dimensional Genomic Structure and Cohesin Occupancy Correlate with Transcriptional Activity during Spermatogenesis. *Cell reports*. **28**: 352–367.e9.

Wang Y et al. 2019. Reprogramming of Meiotic Chromatin Architecture during Spermatogenesis. *Molecular Cell*. **73**: 547–561.e6.

Yin Y, Jiang Y, Lam KWG, Berletch JB, Disteche CM, Noble WS, Steemers FJ, Camerini-Otero RD, Adey AC, and Shendure J. 2019. High-Throughput Single-Cell Sequencing with Linear Amplification. *Molecular Cell*. **76**: 676–690.e10.

Zickler D and Kleckner N. 1999. Meiotic Chromosomes: Integrating Structure and Function. *Annual Review of Genetics*. **33**: 603–754.
